# Retromer Regulates HIV-1 Envelope Glycoprotein Trafficking and Incorporation into Virions

**DOI:** 10.1371/journal.ppat.1004518

**Published:** 2014-11-13

**Authors:** Elisabetta Groppelli, Alice C. Len, Luke A. Granger, Clare Jolly

**Affiliations:** Division of Infection and Immunity, University College London, London, United Kingdom; Fred Hutchinson Cancer Research Center, United States of America

## Abstract

The envelope glycoprotein (Env) of the Human Immunodeficiency Virus Type-1 (HIV-1) is a critical determinant of viral infectivity, tropism and is the main target for humoral immunity; however, little is known about the cellular machinery that directs Env trafficking and its incorporation into nascent virions. Here we identify the mammalian retromer complex as a novel and important cellular factor regulating Env trafficking. Retromer mediates endosomal sorting and is most closely associated with endosome-to-Golgi transport. Consistent with this function, inactivating retromer using RNAi targeting the cargo selective trimer complex inhibited retrograde trafficking of endocytosed Env to the Golgi. Notably, in HIV-1 infected cells, inactivating retromer modulated plasma membrane expression of Env, along with Env incorporation into virions and particle infectivity. Mutagenesis studies coupled with coimmunoprecipitations revealed that retromer-mediated trafficking requires the Env cytoplasmic tail that we show binds directly to retromer components Vps35 and Vps26. Taken together these results provide novel insight into regulation of HIV-1 Env trafficking and infectious HIV-1 morphogenesis and show for the first time a role for retromer in the late-steps of viral replication and assembly of a virus.

## Author Summary

Virus assembly necessitates the hijacking of the host cell machinery in order for new infectious viral particles to be constructed and disseminate. The envelope glycoprotein (Env) of HIV is a critical determinant of viral infectivity and is also a major target for antiviral immune responses. The long cytoplasmic tail of HIV Env plays an essential role in the assembly of infectious virions and limiting exposure of Env to the immune system, but the cellular machinery that transports HIV Env in virus-infected cells remain poorly understood. Here we have identified the mammalian retromer complex involved in endosomal sorting as a novel cellular factor regulating Env trafficking in virus-infected cells. We show that inactivating retromer alters Env localization, cell surface expression and incorporation into virions and that retromer binds directly to the Env cytoplasmic tail to perform these functions. This study defines an important pathway of Env transport and describes for the first time a role for this highly conserved cellular complex in assembly of a virus.

## Introduction

The Human Immunodeficiency Virus type-1 (HIV-1) assembles at the plasma membrane of virus-infected cells from which nascent particles are released by a process of budding. Efficient virus assembly therefore requires correct spatial and temporal trafficking of viral proteins and necessitates critical interactions between viral and cellular cofactors. The envelope glycoprotein (Env) of primate lentiviruses including Human Immunodeficiency Virus type-1 (HIV-1) is a key determinant of viral infectivity, facilitating attachment of virions to the surface of susceptible cells, triggering fusion of the viral and cellular membranes and determining the site of infectious virus assembly at the plasma membrane [1], [2], [3], [4]. Moreover because Env is expressed on the surface of infected cells and is the only viral protein exposed on the virion, it is also the major target for neutralizing antibody responses; however the mechanism of Env trafficking in HIV-1 infected cells and how it is incorporated into viral particles is poorly understood.

HIV-1 Env consists of approximately 856 amino acids and is synthesized in the endoplasmic reticulum as a 160 kDa precursor (gp160). During passage through the secretory pathway, Env undergoes cleavage by the Golgi-localized protease furin [5] to produce two subunits that remain non-covalently associated: the receptor binding surface subunit gp120 and a transmembrane subunit gp41, which are collectively referred to as Env. Gp120 contains the binding sites for the receptor (CD4) and coreceptor (CCR5 or CXCR4) expressed on the surface of susceptible cells and thus determines viral tropism. The gp41 subunit contains an ectodomain, a transmembrane domain, and a cytoplasmic domain that mediates intracellular trafficking, interaction with HIV-1 Gag and incorporation of Env into virions (reviewed in [1]). Notably, the cytoplasmic tail (CT) of Env is long in lentiviruses (around 150 amino acids) by contrast to other retroviruses whose EnvCT is considerably shorter (approximately 50 amino acids)(reviewed in [6]). Conservation of a long CT in HIV-1 suggests the presence of regions, many still undefined, which are critical for efficient viral replication. Indeed, truncation of the cytoplasmic tail of Env has been shown to alter Env intracellular trafficking and to profoundly reduce the infectivity of HIV-1 in many cell types, including CD4 T cells that are the main targets for HIV-1 replication *in vivo* [7], [8].

Following exit from the ER, HIV-1 Env traverses the Golgi complex to the trans-Golgi network (TGN) from where it is trafficked via the secretory pathway to the plasma membrane. Once at the cell surface, Env either interacts with membrane-associated HIV-1 Gag and gets incorporated into viral particles, or alternatively is rapidly endocytosed [9]. Two motifs in the cytoplasmic tail Env can promote efficient internalization: the highly-conserved and well-defined membrane proximal YSPL (YxxL) motif at position 712 in HIV-1 [10], [11], [12] and a C-terminal dileucine sequence [13], either of which can act autonomously to mediate clathrin-dependent endocytosis. The fact that the endocytic motifs are highly-conserved across HIV-1 strains and related simian lentiviruses suggests that efficient internalization of Env from the plasma membrane plays an important role in the viral life-cycle. However, once within the endosomal pathway the intracellular itinerary of Env and how trafficking is regulated remains unclear.

Retromer is a cellular protein complex and member of the endosomal sorting machinery that is conserved from yeast [14] to humans [15], [16], [17], [18] and plays an essential role in endosomal sorting of a select group of physiologically important cargo proteins. Retromer operates in eukaryotes by recognizing and sorting cargos in maturing endosomes into nascent endosomal tubules, and is most closely associated with retrograde transport back to the Golgi complex [17], [18], [19], [20]. Increasing evidence also points to a role for retromer in the transport of some cargo proteins from endosomes to the plasma membrane [19], [21], [22]. Mammalian retromer is a heteropentameric complex comprising a cargo-selective trimer of Vps26-Vps29-Vps35 and a membrane-bending complex consisting of two sorting nexins (SNX1 or SNX2 with SNX5 or SNX6) (reviewed in [19], [20], [23]). Selection of cargo proteins for retromer-mediated trafficking is by direct binding to Vps35 or Vps26 [17], [24], [25], [26], [27], [28] and depleting either ablates retromer function. One of the best characterized cargos for retromer sorting is the cation-independent mannose 6-phosphate receptor (CIMPR), a membrane protein whose primary function is to transport newly synthesized acid hydrolases from the TGN to endosomes for eventual delivery to lysosomes. Following dissociation of their cargos in endosomes, CIMPRs are recycled via retrograde transport to the TGN in a retromer-dependent manner, thus avoiding lysosomal degradation [17], [18], [25]. In the absence of retromer, CIMPRs fail to retrieve to the TGN and are missorted to the plasma membrane and/or degraded in lysosomes [17], [18], [25]. A number of other proteins are also trafficked in mammalian cells in a retromer-dependent manner [26], [29], [30], [31], [32] and collectively these studies have placed the retromer complex at the center of endosomal sorting.

Based on the known role of retromer in endosomal sorting and the fact that HIV-1 Env traverses the endosomal network in HIV-1 infected cells, we hypothesized that retromer may be implicit in Env trafficking. Here we report that the mammalian retromer complex is an important cellular cofactor regulating the intracellular transport of Env. We found that inhibiting retromer function relocalized Env in both HIV-1 infected and Env expressing cells, resulting in increased plasma membrane expression, greater incorporation into virions and failure of endocytosed protein to retrieve to the Golgi. Notably, this was dependent on the gp41 cytoplasmic tail of Env that we show binds directly to retromer. These findings identify retromer as a novel modulator of HIV-1 Env transport and infectious HIV-1 assembly and describe for the first time a role for the mammalian retromer complex in assembly of a virus.

## Results

### Inactivating retromer modulates Env trafficking in HIV-1-infected cells and incorporation into viral particles

**Figure 1:**
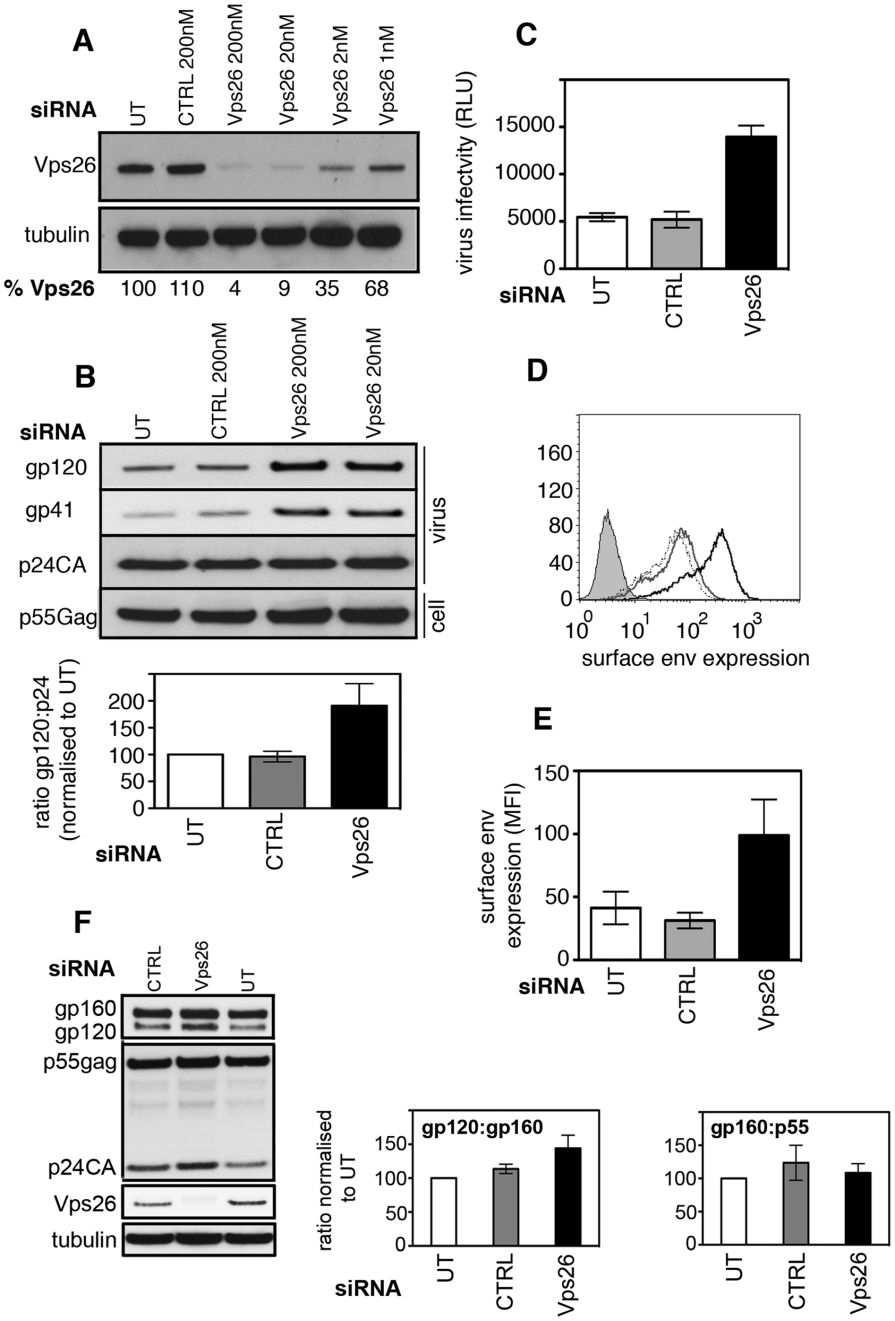
Vps26 depletion increases plasma membrane expression of HIV-1 Env and incorporation into viral particles. **A)** HeLa TZM-bl cells were transfected twice with control or Vps26 siRNA. Forty-eight hours after the second transfection cell lysates were prepared and subjected to SDS-PAGE and western blotting for Vps26 and actin as a loading control. Band intensities were quantified using ImageJ and the percentage of remaining Vps26 relative to actin was calculated. One representative Western blot from three independent experiments is shown. **B)** HeLa TZM-bl cells were infected with HIV-1 immediately after the second knockdown. Forty-eight hours later viral supernatants were pelleted by ultracentrifugation through sucrose and subjected to SDS-PAGE and western blotting for HIV-1 Env (gp120 and gp41) and Gag (p24CA). One representative Western blot from three independent experiments is shown. The band intensities from three independent experiments were quantified using ImageJ and the ratio of HIV-1 Env gp120 to Gag p24CA is shown. Error bars show the SEM. Cell lysates were also prepared and blotted for Gag p55 (lower panel). **C)** Equal volumes of viral supernatants from infected cells were used to infect Jurkat 1G5 reporter cells. Infectious virus titer is shown as luciferase relative light units (RLU). One representative of three independent experiments is shown. Errors bars show the SEM. **D)** Flow cytometry analysis of HIV-1 Env plasma membrane expression following Vps26 KD. One histogram representative of three independent experiments is shown. Cells were either left uninfected (filled grey), untreated and infected (solid grey line), control siRNA treated and infected (broken black line) or Vps26 siRNA treated and infected (solid black line) and surface stained for Env on ice prior to fixation. **E)** Pooled cell surface mean fluorescence intensity (MFI) of Env staining from three independent experiments. Error bars show the SEM. **F)** Cell lysates from HIV-1 infected siRNA treated cells were subjected to SDS-PAGE and Western blotting for Vps26, tubulin, HIV-1 Env and Gag. One representative of three independent experiments is shown. The band intensities from three independent experiments were quantified using ImageJ and the ratio of unprocessed (gp160) and processed (gp120) Env and Gag (p55) is shown. Error bars show the SEM.

To investigate whether the mammalian retromer complex plays a role in HIV-1 replication, sequential rounds of siRNA transfection were used to deplete HeLa TZM-bl cells of Vps26A, an essential component of the retromer complex. Vps26A is the predominant isoform of Vps26 in HeLa cells, constituting >90% of the total Vps26 [33] and depletion of Vps26A ablates retromer function by abrogating the function of the cargo selective trimer complex [17], [18], [20], [25]. Western blotting confirmed that endogenous Vps26A (hereafter referred to as Vps26) was readily detected in untreated HeLa cells and in cells transfected with control siRNA, but was almost completely depleted after two rounds of transfection with siRNA targeting Vps26 (96% and 91% reduction in Vps26 at 200 nM and 20 nM respectively) (Figure 1A). As expected, further titration of the siRNA to 2 nM and 1 nM gave less efficient depletion of Vps26 (Figure 1A) therefore higher concentrations of siRNA were used in all subsequent experiments. In agreement with previous reports [17], [18], [25], we observed that Vps26 knockdown redistributed a proportion of intracellular CIMPR to early endosomes and increased colocalization with EEA1, thereby confirming phenotypic loss of function (R value for CIMPR/EEA1  = 0.34+/−0.02 in untreated cells and 0.44+/−0.02 in Vps26 knockdown cells, *p* = 0.007) (Figure S1).

To test the consequences of inactivating retromer for HIV-1 replication, HeLa TZM-bl cells (that express the HIV-1 entry receptors CD4, CXCR4 and CCR5 and contain an HIV-1-Tat inducible luciferase reporter gene) were transfected with siRNA targeting Vps26 and infected with the replication competent HIV-1 strain NL4.3 immediately after the second knockdown. Twenty-four hours later luciferase activity was measured to quantify virus infection. This short time interval meant that we were measuring the early steps of infection. No difference in reporter gene expression was detected between control and Vps26-depleted cells, demonstrating that Vps26 knockdown did not inhibit viral entry or the early steps of HIV-1 infection (Figure S2A). Furthermore, western blotting of cell lysates showed that HIV-1 infected cells (>95% of cells HIV-1 Gag+) and uninfected cells expressed similar levels of Vps26 and Vps35 protein, demonstrating that HIV-1 infection alone does not modulate retromer expression (Figure S2B and C). To determine whether retromer knockdown affected HIV-1 assembly and budding, virus-containing supernatants were harvested from HIV-1 infected cells at 48 h post-infection, purified by ultracentrifugation and equal volumes of purified virions were subjected to SDS-PAGE and western blotting for viral proteins (Figure 1B). Probing membranes with antisera specific for HIV-1 Gag revealed similar levels of Gag p24 in viral supernatants harvested from control and Vps26 siRNA treated cells (*p*>0.05 quantified from four independent experiments). Similar levels of Gag precursor p55 were also present in cell lysates (*p*>0.05) showing that inactivating retromer had no effect on Gag expression, processing or virus budding. Strikingly, when the same blots were probed with antisera against HIV-1 Env we observed clear increase in the amount of Env gp120 and gp41 incorporated into virions produced by Vps26 KD cells. Quantification of western blots from three independent experiments showed that Vps26 KD consistently resulted in a 2−3 fold increase in the amount of Env gp120 in viral particles relative to Gag p24. To determine whether the increased Env content correlated with increased infectivity, viral supernatants were used to infect fresh HeLa TZM-bl cells and infection was measured by luciferase assay. Consistent with the increased Env incorporation, virions produced by Vps26KD cells displayed a 2−3 fold increase in particle infectivity, confirming that the extra gp120 packaged in virions was functional (Figure 1C).

Env is incorporated into nascent particles as the viral cores bud through the plasma membrane. We therefore reasoned that the greater Env content of virions might reflect increased plasma membrane expression on cells depleted of Vps26. To test this, virus-infected cells were stained on ice with an anti-Env antibody to label only cell surface exposed protein and analyzed by flow cytometry. Figures 1D and E show that Vps26 KD cells expressed 3 fold more Env on the cell surface when compared to untreated or control siRNA treated cells. Western blotting of total cell lysates prepared from HIV-1 infected cells treated with Vps26 or control siRNA revealed no statistically significant difference in the relative abundance of Env gp160 (gp160:gp120 ratio, *p* = 0.08 from 4 independent experiments) and no difference in the gp160 to Gag p55 ratio (*p* = 0.1) (Figure 1F). From these data we conclude that changes to Env synthesis or proteolytic processing of gp160 are unlikely to account for the increased Env content of virions. Taken together these data suggest a specific role for retromer in HIV-1 Env trafficking and its subsequent incorporation into nascent viral particles.

### HIV-1 Env partially colocalizes with retromer in virus-infected cells

**Figure 2:**
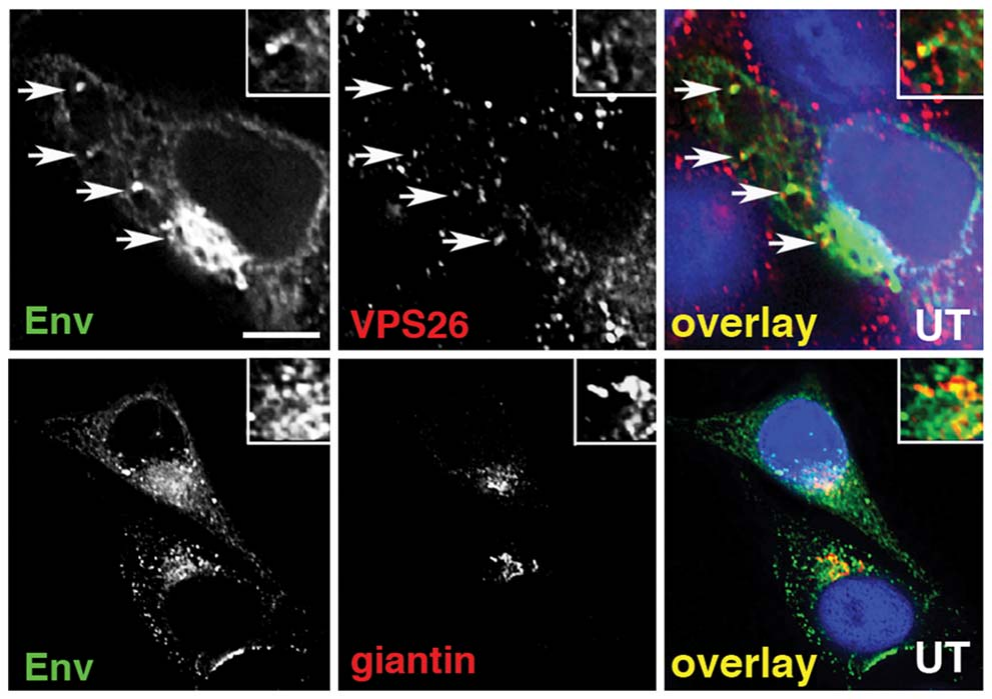
Immunofluorescence staining of Env in HIV-1 infected cells. **A)** HeLa TZM-bl cells were infected with HIV-1, fixed, permeabilized and stained for HIV-1 Env (green) and retromer component Vps26 or the Golgi marker giantin or (red). Panels are single *xy* slices and are representative examples from three independent experiments. Arrows highlight coincident labeling of Env and Vps26. Scale bar is 20 microns. The amount of immunoreactive Env colocalizing with Vps26 or giantin was calculated from at least 20 cells.

To further investigate retromer-dependent Env sorting, we performed immunofluorescence microscopy to examine the steady-state localization of Env in virus-infected cells. Cells infected with HIV-1 were fixed, permeabilized and stained for Env (mAb 2G12), the Golgi (giantin) and the retromer component Vps26. Intracellular staining revealed that a proportion of the total immunoreactive Env colocalized with the retromer component Vps26 in HIV-1 infected cells (R value for Env/Vps26  = 0.34+/−0.03) (Figure 2A). This was most clearly seen when examining peripheral Env + cytoplasmic foci. As expected, we also observed that in untreated cells the majority of intracellular Env was concentrated in a perinuclear region and colocalized with the Golgi marker giantin (R value for Env/giantin  = 0.52+/−0.02). That we observed a partial colocalization of Env and retromer is indicative of the dynamic nature of Env trafficking through the endosomal network. Vps26 knockdown did not induce any apparent accumulation of Env in early endosomes or Lamp1 + lysosomes (Figure S3), indicating that Env was not sequestered into intracellular compartments, but rather (as the flow cytometry data suggest) that surface expression was increased after Vps26 knockdown. In agreement with the data showing no effect of Vps26 KD on HIV-1 Gag budding or processing (Figure 1), a punctate cytoplasmic pattern of Gag staining was observed with little or no overlap with Vps26 (Figure S4).

### The gp41 cytoplasmic tail of Env determines sensitivity to Vps26 depletion

**Figure 3:**
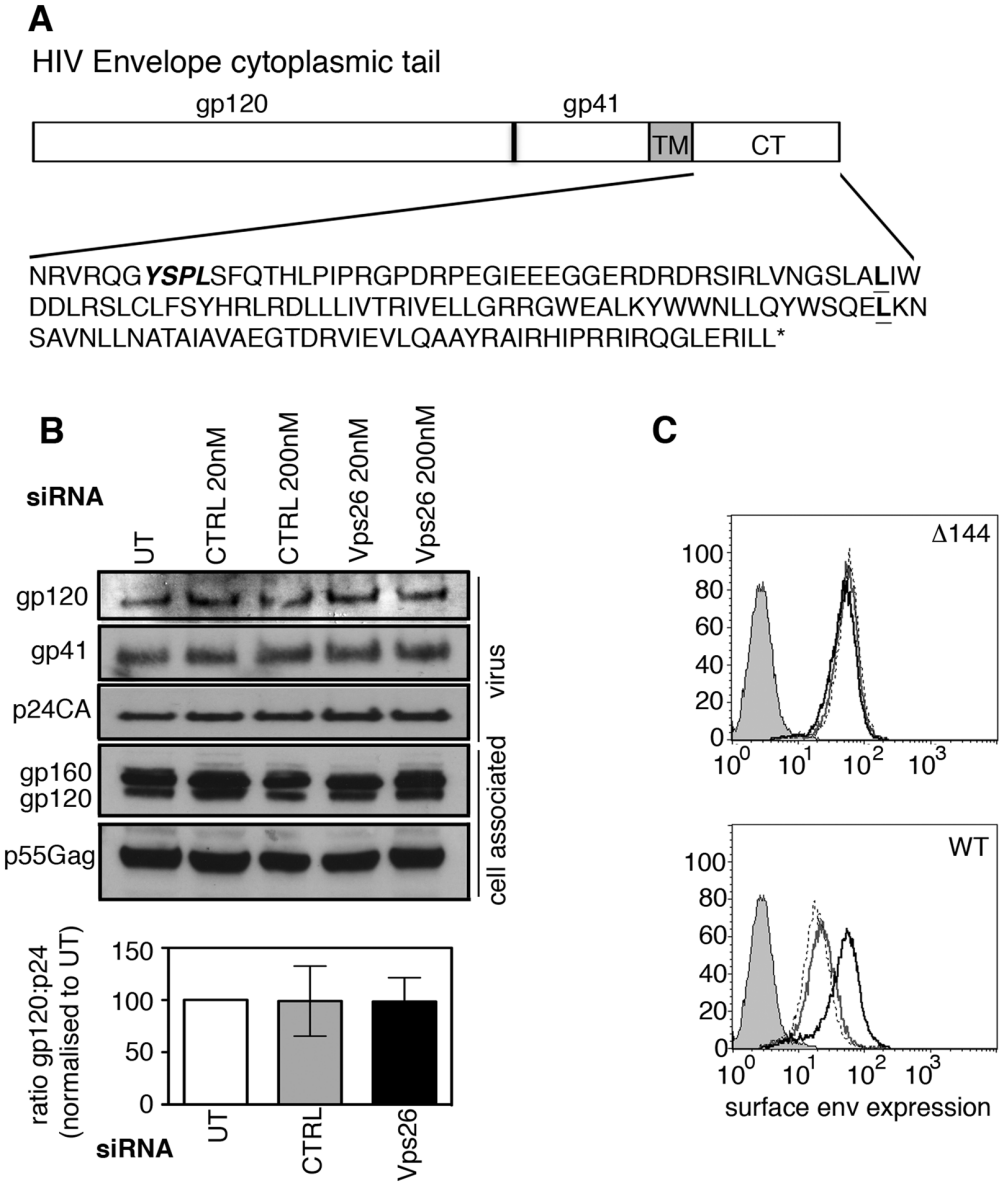
The cytoplasmic tail of HIV-1 Env mediates sensitivity to retromer-depletion. **A)** Amino acid sequence of the NL4.3 HIV-1 cytoplasmic tail. YSPL in bold italics highlights the membrane-proximal YxxL endocytosis motif. The two leucines in bold show the site of truncations of the gp41 cytoplasmic tail in the CD8-gp41 constructs used in Figure 6. **B)** HeLa TZM-bl cells were transfected with control or Vps26 siRNA and infected with Δ144 NL4.3 lacking the C-terminal 144 amino acids of the Env cytoplasmic tail. Viral supernatants were pelleted and cell lysates prepared and subjected to SDS-PAGE and Western blotting for HIV-1 Env and Gag. One representative western blot from three independent experiments is shown. The band intensities from three independent experiments were quantified using ImageJ and the ratio of HIV-1 Env gp120 to Gag p24CA in virus preparations is shown. Error bars show the SEM. **C)** Flow cytometry analysis of HIV-1 Env plasma membrane expression following Vps26 KD. Cells were either left uninfected (filled grey), untreated and infected (solid grey line), control siRNA treated and infected (broken black line) or Vps26 siRNA treated and infected (solid black line). One representative from three independent experiments performed with HIV-1 Δ144 (top panel) and WT (lower panel) is shown.

Because HIV-1 Env is a transmembrane protein, the cytoplasmic tail projects through cellular membranes into the cytosol and is available to interact with components of the cellular trafficking and endosomal sorting machinery, such as retromer that resides on the cytosolic face of endosomal membranes [34], [35]. The cytoplasmic tail of the gp41 subunit of HIV-1 Env (gp41CT) is relatively long, spanning residues 706_HxB2_ to 856_HxB2_ (corresponding to 704_NL4.3_ to 854_NL4.3_) (Figure 3A). To investigate whether retromer-mediated Env trafficking is dependent on the gp41CT, we used a mutant of the HIV-1 strain NL4.3 that contains a 144 amino acid deletion in the cytoplasmic tail, leaving only the first 6 residues proximal to the transmembrane domain [36], [37] (referred to herein as NL4.3 Δ144) (Figure 3A). This virus retains infectivity in many commonly used cell lines [7], [8], [13], [37] including HeLa TZM-bl cells. In direct contrast to what was observed using WT virus containing the complete gp41CT (Figure 1 and Figure 3C), infecting cells depleted of Vps26 with NL4.3 Δ144 did not result in an increase in Env expression at the cell surface or alter the amount of gp120 and gp41 incorporated into virions (Figures 3B and 3C). These data show that deletion of the Env gp41CT renders HIV-1 completely insensitive to retromer depletion, suggesting that the cytoplasmic tail of Env is a critical determinant of retromer-dependent sorting. It should be noted that consistent with previous reports, untreated cells infected with NL4.3 Δ144 expressed higher levels of Env on plasma membrane compared to cells infected with WT virus, owing to deletion of endocytic signals in the cytoplasmic tail (Figure 3C).

### Retromer promotes Golgi retrieval of endocytosed Env

**Figure 4:**
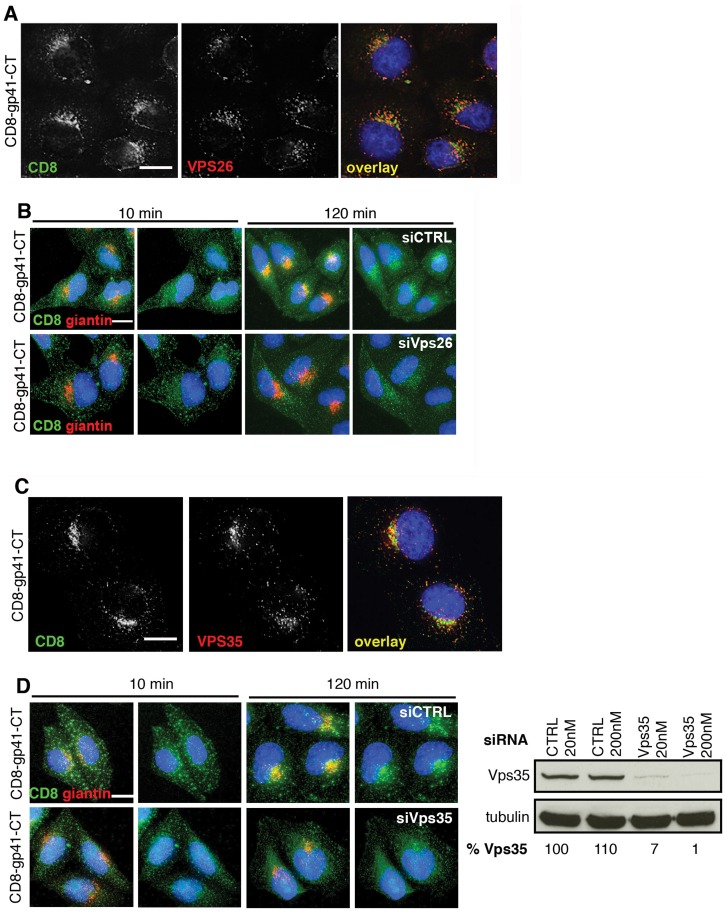
The gp41 cytoplasmic tail mediates retromer-dependent Golgi retrieval. **A)** Immunostaining of intracellular CD8-gp41CT (green) and Vps26 (red) in HeLa cells. Panels are single *xy* slices and are a representative example from three independent experiments. Coincident staining appears yellow. Scale bar is 10 microns. **B)** CD8-gp41CT expressing cells were treated with control or Vps26 siRNA. Cells were incubated with anti-CD8 monoclonal antibody at 4°C for 30 min, washed and incubated 37°C for 10 and 120 min. Cells were fixed, permeabilized and stained for the Golgi marker giantin (red) and fluorescently conjugated anti-mouse secondary antibody to localize the pool of internalized CD8-gp41CT (green). Panels are maximum intensity projections reconstructed from serial *Z* sections through the entire volume of the cell. Data are representative of three independent experiments. Scale bar is 20 microns. **C)** Immunostaining of total intracellular CD8-gp41CT (green) and Vps35 (red) in HeLa cells. Panels are single *xy* slices and are a representative example from three independent experiments. Coincident staining appears yellow. Scale bar is 15 microns. **D)** CD8-gp41CT expressing cells were treated with control or Vps35 siRNA and antibody-feeding and Golgi retrieval performed as described in B. Data are representative of three independent experiments. Scale bar is 20 microns. Western blot confirming efficient knockdown of Vps35 in HeLa cells following transfection with control or Vps35 siRNA as described in Figure 1. Band intensities were quantified using ImageJ and the percentage of remaining Vps35 relative to tubulin was calculated. One representative Western blot from three independent experiments is shown.

To further define the role of the Env gp41CT and to interrogate retromer-dependent trafficking of Env in the absence of other viral proteins, we generated reporter constructs in which the ecto and transmembrane domains of CD8 (that contain no known trafficking motifs) were cloned upstream of the Env gp41CT to generate chimeric CD8-gp41 reporter proteins. Constructs were made that contained the entire HIV-1 Env gp41CT (CD8-gp41-CT) or the CIMPR cytoplasmic tail as positive control. Stable cell lines were generated and expression validated by western blotting (Figure S5). Immunofluorescence staining of cells stably expressing reporter constructs revealed that the full-length gp41CT (CD8-gp41CT) colocalized with vesicular Vps26 (R value  = 0.3+/−0.03) (Figure 4A), showing that the cytoplasmic tail of Env is alone sufficient for trafficking of Env through the retromer-containing compartment.

The mammalian retromer complex is associated with retrograde transport of a select group of cargo proteins from endosomes to the Golgi complex [19], but because most steady-state Env is present within the Golgi complex (Figure 2) [13], [38] specific endosome-to-Golgi recycling can be difficult to discern when staining steady-state intracellular protein. Especially so when there is newly synthesized protein moving through the Golgi. Therefore to determine whether retromer mediates endosomal sorting and Golgi retrieval of Env, we performed an antibody-feeding assay to specifically follow trafficking of CD8-gp41CT after endocytosis from the plasma membrane. A similar approach has been taken to show the involvement of retromer in the retrieval of a CD8-CIMPR reporter [18] and to identify a sorting motif in the CIMPR tail [25]. Surface staining for CD8 and flow cytometry analysis confirmed that CD8-gp41CT was expressed at the plasma membrane (MFI  = 150.9+/−20.3 compared to 10.8+/−0.5 for untransfected cells). HeLa cells expressing gp41CT constructs were incubated on ice with anti-CD8 in order to label surface exposed protein, washed extensively and incubated at 37°C to allow endocytic uptake and intracellular trafficking. We confirmed that non-specific fluid-phase uptake of antibody was undetectable using cells that did not express CD8 fusion proteins (Figure S6). Figure 4B shows that following incubation at 37°C, endocytosed CD8-gp41CT protein relocalized from a generally peripheral and punctate cytoplasmic distribution at 10 min, to a more defined perinuclear region at 120 min and showed increased colocalization with the Golgi marker giantin, indicative of gp41CT-mediated endosome-to-Golgi retrieval (CD8/giantin R value at 10 min  = 0.26+/−0.02 and at 120 min  = 0.39+/−0.03). Notably, in cells depleted of Vps26 the endocytosed protein displayed a more vesicular intracellular staining pattern, with weaker perinuclear localization and giantin co-staining (R value at 120 min  = 0.29+/−0.02, *p* = 0.03). The same results were obtained when RNAi was also used to deplete HeLa cells of Vps35 that also abrogates retromer function [17]. As expected we found that steady-state CD8-gp41CT colocalized with Vps35 in untreated cells (Figure 4C). Importantly, Vps35 KD impaired endosome-to-Golgi retrieval of endocytosed CD8-gp41CT, in exact agreement with what we observed following Vps26 KD (Figure 4D, CD8/giantin R value at 120 min for CTRL  = 0.42+/−0.01; Vps35 siRNA  = 0.33+/−0.03, *p* = 0.001). Taken together, these data show that two independent routes to retromer inactivation result in impaired endosome-to-Golgi retrieval of Env, providing compelling evidence for a specific role for retromer in retrograde trafficking of Env.

### Retromer interacts directly with the EnvCT

**Figure 5:**
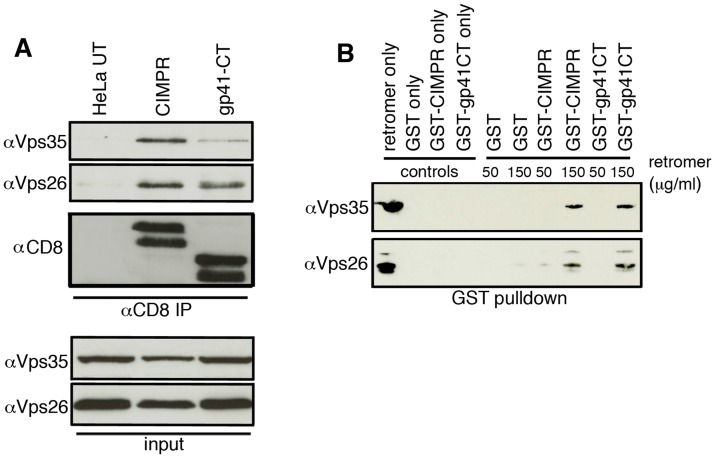
The gp41 cytoplasmic tail binds directly to retromer. **A)** Native coimmunoprecipitation with anti-CD8 identifies retromer components Vps26 and Vps35 as interacting with the HIV-1 gp41CT. Cell lysates prepared from untransfected (UT), CD8-CIMPR or CD8-gp41CT expressing HeLa cells were incubated with anti-CD8 coated beads and co-IP proteins were subjected to SDS-PAGE and western blotting for Vps35 and Vps26. Untransfected HeLa cells were used as a negative control and CD8-CIMPR as a positive control. **B)** GST-pulldown confirms direct binding of the gp41CT to retromer. Purified recombinant FLAG-tagged retromer complex (3xFLAG-Vps26-Vps29-Vps35-His_6_) was incubated with purified GST or GST fusion proteins containing the CIMPR (GST-CIMPR) or Envgp41 (GST-gp41CT) cytoplasmic tail and proteins were pulled down with glutathione-Sepharose 4B beads. Bound retromer components Vps26 and Vps35 were detected by immunoblotting.

Having shown that retromer mediates Golgi recycling in a gp41CT-dependent manner, we next sought to determine whether the gp41CT physically interacts with retromer using native coimmunoprecipitation (co-IP). Lysates prepared from HeLa cells stably expressing CD8-gp41CT, CD8-CIMPR protein or untransfected controls were incubated with anti-CD8 coated beads and immunoprecipitated proteins were analyzed by western blotting for Vps35 and Vps26 (Figure 5A). As expected, the CD8-CIMPR included as a positive control readily coimmunoprecipitated both Vps35 and Vps26 (Figure 5A) [18], [25]. Importantly, the CD8-gp41CT protein was also able coimmunoprecipitate both Vps35 and Vps26 indicative of an interaction between the EnvCT and the retromer complex. Mass spectrometry analysis of bands excised from coomassie stained gels confirmed the presence of Vps26 in samples immunoprecipitated from both CD8-CIMPR and CD8-gp41CT expressing cells, but not in untransfected cells (Figure S7).

In order to determine if binding of the gp41CT to the retromer complex was direct, GST pulldown assays were performed. Bacterially expressed GST-gp41CT was purified and incubated with a bacterially expressed and purified FLAG-tagged retromer complex, which consists of the cargo selective trimer components 3xFLAG-Vps26-3x-FLAG-Vps29-3x-FLAG-Vps35-His_6_ [26]. GST only was used as negative control and GST-CIMPR as a positive control. Western blotting for retromer components using antibodies specific for Vps26 and Vps35 showed that gp41CT specifically immunoprecipitated retromer in a dose-dependent manner, as did the GST-CIMPR, while GST alone did not pulldown retromer (Figure 5B). Immunoreactive Vps26 and Vps35 were also detected in the lanes loaded with the purified retromer complex only. Taken together, these results show that the cytoplasmic tail of Env physically interacts with retromer and that binding is direct and does not require additional cellular factors.

### The C-terminal 100 amino acids of the gp41CT are necessary for retromer binding and Golgi retrieval

**Figure 6:**
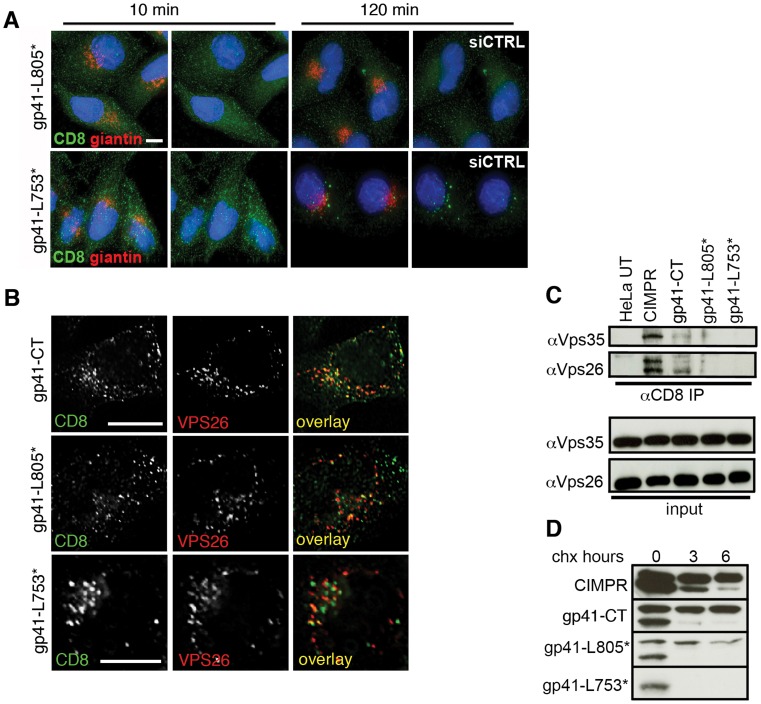
The C terminal 100 amino acids of the Env cytoplasmic tail are required to bind retromer. **A)** Cells expressing reporter constructs containing truncated EnvCT were treated with control or Vps26 siRNA and an antibody feeding assay was performed to follow Golgi retrieval of endocytosed protein as described in Figure 4. Golgi marker giantin (red) and internalized CD8-gp41CT (green). Panels are maximum intensity projections reconstructed from serial *Z* sections through the entire volume of the cell. Data are representative of three independent experiments. Scale bar is 20 microns. **B)** Cells from A stained for Vps26 show that full length and truncated mutants are all efficiently endocytosed and colocalize with intracellular Vps26. Scale bar is 20 microns. **C)** Truncation of the gp41CT abrogates co-IP of Vps26 and Vps35. Cell lysates prepared from untransfected (UT) or cells stably expressing CD8-reporter constructs were incubated with anti-CD8 coated beads and co-IP proteins were subjected to SDS-PAGE and Western blotting for Vps35 and Vps26. Untransfected HeLa cells were used as a negative control and CD8-CIMPR as a positive control. **D)** HeLa cells expressing CD8-reporter constructs were treated with cycloheximide for the indicated periods of time and cell lysates subjected to SDS-PAGE and western blotting. One representative of two independent experiments is shown.

To further define the role of the gp41CT in retromer-dependent Env trafficking, endocytic uptake and Golgi retrieval assays were performed using constructs in which the C terminal 50 or 100 amino acids of gp41 were deleted (CD8-gp41-L805* and CD8-gp41-L753* respectively) (Figure S5). Figure 6A shows that although both the CD8-gp41-L805* and CD8-gp41-L753* proteins were readily detected after 10 minutes of endocytic uptake at 37°C, they showed markedly reduced immunofluorescence staining after 120 minutes and failed to retrieve to the Golgi complex (gp41-L805* and giantin R value at 120 min  = 0.21+/−0.02; gp41-L753* and giantin R value at 120 min  = 0.20+/−0.04). Colocalization of all CD8-gp41 proteins with intracellular Vps26 was readily detected after 10 minutes incubation at 37°C (R values for CD8 and Vps26 for: gp41CT  = 0.48+/−0.1; gp41-L805*  = 0.35+/−0.04; gp41-L753*  = 0.46+/−0.07) (Figure 6B), demonstrating that failure to recycle to the Golgi was not due to a block in endocytic uptake of these truncation mutants. This is in agreement with published data showing that cargos that can no longer interact with retromer may still colocalize with Vps26-containing intracellular compartments [25].

Having shown that truncated gp41CT mutants were impaired in recycling to the Golgi after endocytic uptake, we sought to determine if these proteins were no longer able bind retromer. Whereas the CIMPR and gp41CT were both able to pull-down retromer components Vps26 and Vps35 by co-IP, CD8-gp41-L753* containing the largest C- terminal deletion repeatedly failed to co-IP either Vps35 or Vps26 (Figure 6C). Barely detectable bands corresponding to Vps35 and Vps26 were seen in immunoprecipitates from cells expressing the CD8-gp41-L805*. We observed that the truncated gp41CT proteins that failed to bind retromer and did not retrieve to the Golgi complex also showed a gradual loss of immunofluorescence staining, suggestive of reduced protein stability. To investigate this, cycloheximide was used to arrest protein synthesis and degradation of existing protein was followed over time by western blotting (Figure 6D). As previously described, the CD8-CIMPR migrates as a doublet during SDS-PAGE [25]. In agreement with previous reports [25] we observed that the higher molecular weight CIMPR band remained relatively stable over time in the presence of cycloheximide (95% remaining at 6 h when normalized to 0 h = 100%). Notably, similar results were seen for the full length CD8-gp41CT that also migrated as a doublet, with the higher molecular weight band appearing stable and readily detected up to 6 h after cycloheximide treatment (99% of protein remaining at 6 h). By contrast CD8-gp41-L805* showed more rapid degradation of the higher molecular weight protein (68% remaining at 6 h). The short CD8-gp41-L753* protein (that showed the most marked loss of immunofluorescence staining after extended incubation at 37°C) migrated only as the lower molecular weight species that was rapidly lost in all samples. These data reveal that CD8-gp41CT proteins that fail to bind to retromer (by co-IP) and do not recycle to the Golgi appear to be less stable.

**Figure 7:**
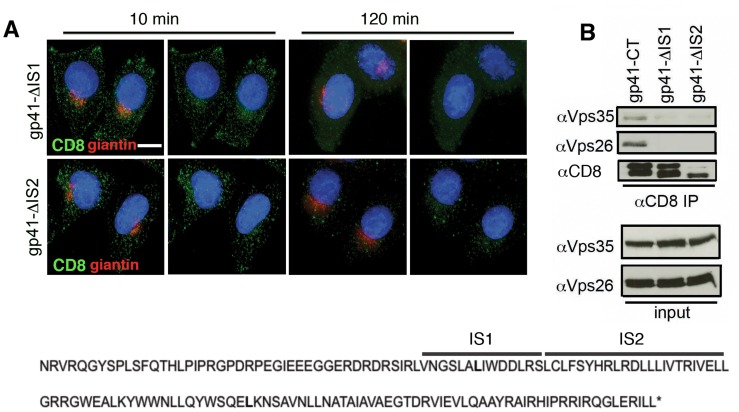
Identification of two internal sequences within the EnvCT required for retromer binding. **A)** Antibody-feeding and retrieval assay was performed as described in Figure 4. Panels are maximum intensity projections reconstructed from serial *Z* sections through the entire volume of the cell. Data are representative of two independent experiments. Scale bar is 20 microns. **B)** Cell lysates prepared from cells stably expressing CD8-gp41CT constructs were incubated with anti-CD8 coated beads and co-IP proteins were subjected to SDS-PAGE and Western blotting for Vps35 and Vps26. Deleting either of two internal regions of the EnvCT abrogates co-IP of Vps26 and Vps35.

It has previously been reported that two adjacent internal regions within the EnvCT may be involved in trafficking Env back to the Golgi complex [39] and influence Env incorporation into virions [40]. To test whether these regions were implicit in retromer-dependent Env sorting, site-directed mutagenesis was used to independently delete these sequences from the CD8-gp41CT protein. The first mutant (termed Δis1) has an internal deletion that removes a 14 amino acid stretch V747-S760 (inclusive of S760). The second mutant (termed Δis2) has an internal deletion that removes 23 amino acids from L761-L783 (inclusive of L783). Both mutants remove amino acids immediately downstream of the CD8-gp41-L753* truncation. Figure 7A and B show that deletion of either is1 or is2 resulted in loss of binding of the EnvCT to retromer and failure to coimmunoprecipitate Vps26 and Vps35. Moreover, both mutants failed to retrieve to the Golgi following endocytosis from the plasma membrane (R values for CD8 and giantin at 120 min: gp41CT  = 0.44+/−0.05; Δis1 = 0.1+/−0.06; Δis2 = 0.08+/−0.07).

### Retromer modulates Env trafficking in HIV-1 infected T cells

**Figure 8:**
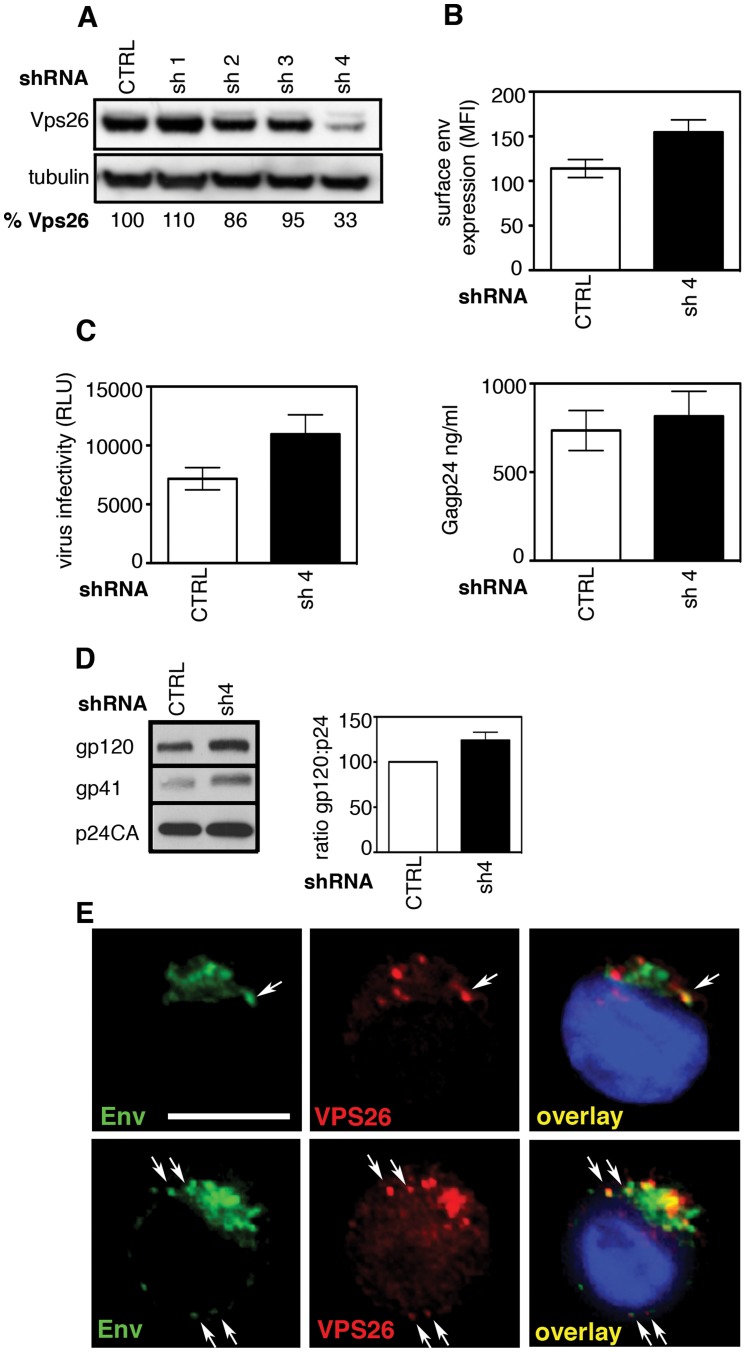
The effect of retromer depletion on HIV-1 assembly in T cells. **A)** Western blot analysis of Vps26 expression in Jurkat T cells stably expressing shRNA targeting Vps26 or control non-targeting shRNA. Quantification relative to tubulin was performed using ImageJ. **B)** Flow cytometry analysis of cell surface Env expression on Jurkat cells stably expressing control or Vps26 targeting shRNA following HIV-1 infection. **C)** Equal volumes of supernatants harvested from HIV-1 infected cells were used to infect reporter cells and HIV-1 infectivity was measured by luciferase assay (RLU, relative light units) (left panel) and Gag p24 was quantified by ELISA to measure the viral content of supernatants (right panel). **D)** Western blotting for Env (gp120 and gp41) and Gag (p24CA) in virions purified from Jurkat T cells expressing control or Vps26 shRNA. The band intensities from two western blots were quantified using ImageJ and the ratio of HIV-1 Env gp120 to Gag p24CA is shown. **E)** HIV-1 infected Jurkat cells were stained for Env (green) and Vps26 (red). DAPI is blue. Scale bar is 10 microns.

CD4 T cells are the main targets for HIV-1 replication. To investigate the effect of retromer depletion in these cells we generated Jurkat T cell lines stably expressing lentiviral shRNA against Vps26 or a non-targeting control sequence. Western blotting identified one hairpin (designated sh-4) that reduced Vps26 expression in T cells by 67% (Figure 8A). When infected with HIV-1, cells expressing sh-4 were found to express 50% more Env at the cell surface compared to cells that were transfected with a non-targeting shRNA control (Figure 8B). Furthermore, analysis of particle infectivity revealed that virions produced from Vps26-depleted Jurkat cells were 50% more infectious on a per particle basis (Figure 8C) and that there was no effect on HIV-1 Gag budding (Figure 8C). Altered Env incorporation into virions was also evident in Vps26 KD cells (Figure 8D). Immunofluorescence staining showed that Env also partially colocalized with Vps26 in a perinuclear region of HIV-1 infected Jurkat cells (Env/Vps26 R value  = 0.5+/−0.05)(Figure 8E); however the comparatively small cytoplasm of T cells makes colocalization analysis considerably more difficult than HeLa cells. Thus, although the efficiency of Vps26 knockdown in T cells was more modest than can be achieved in HeLa cells, the effect on Env was proportional and supports a role for retromer in Env trafficking in T cells.

## Discussion

The assembly of HIV-1 virions takes place at the plasma membrane of infected cells during which Env is incorporated into nascent particles as the viral capsid buds through the cell membrane. Although Env is critical for viral infectivity, the cellular factors regulating Env transport in HIV-1 infected cells and thus its incorporation into nascent virions are ill defined. Most steady-state Env is localized to the Golgi complex, not only because Env traverses the Golgi en route to the plasma membrane, but also because Env that is not incorporated into virions can be recycled back to the Golgi following endocytosis [10], [11], [12], [13]. Based on the known cellular function of retromer in endosomal sorting and Golgi retrieval, we hypothesized that the mammalian retromer complex may play a key role in HIV-1 Env trafficking. Here we have tested this and coupled RNAi with functional virology, trafficking assays and coimmunoprecipitations and find that retromer regulates intracellular trafficking of Env and promotes retrograde endosome-to-Golgi transport, in a manner that is dependent on a direct interaction between the Env cytoplasmic tail and the retromer complex. Perturbing retromer-mediated trafficking of Env, using either RNAi or deleting domains within the EnvCT resulted in failure of endocytosed Env to retrieve to the Golgi complex. In HIV-1 infected cells, subsequent missorting of Env following retromer depletion altered the localization of Env, which in turn impacted on plasma membrane expression and incorporation of Env into nascent viral particles. Taken together, these results identify retromer as a novel cellular factor regulating HIV-1 Env trafficking and infectious virus assembly.

Once cargo proteins are endocytosed from the plasma membrane and reach the endosome, they may either continue along the endosomal maturation pathway and be delivered to lysosomes where they are degraded, or be directed into recycling compartments for trafficking back to the plasma membrane, or be sorted back to the Golgi complex. Retromer is believed to function by sorting a select group of cargo proteins into tubular endosomal membranes and is most-closely associated with the pathway of endosome-to-Golgi transport [19], [33]. Although recent work has revealed retromer also operates in transporting some cargo proteins from endosomes to the plasma membrane [19], [21], [22], our data suggest that in the case of HIV-1 Env, retromer functions to transport Env via the endosome-to-Golgi route since ablating retromer function did not reduce Env surface expression. Our results demonstrating retromer-dependent Golgi retrieval of endocytosed Env are consistent with previous studies that have implicated other cellular factors that intersect the Golgi retrieval pathway in HIV-1 replication, including Rab9 and its interactors p40 and PIKfyve, as well as TIP47 [38], [41], [42], although the function of TIP47 in endosome-to-Golgi transport and its contribution to Env trafficking remains unclear [43], [44], [45]. Furthermore, the clathrin adaptor AP-1 that can promote transport of proteins between endosomes and the Golgi also binds to the cytoplasmic tail of Env [10], [46], [47]. The association of Env with multiple cellular factors that are implicit in endosomal sorting and retrograde transport suggests that this pathway may play an important role during HIV-1 replication, possibly by protecting endocytosed protein from degradation by bypassing trafficking to lysosomes; limiting Env exposure to immune surveillance and humoral immunity by regulating cell surface expression and virion incorporation [48]; or by providing repeated exposure of Env to necessary Golgi modifications.

In addition to blocking endosome-to-Golgi transport, we found that inactivating retromer in HIV-1 infected cells was associated with increased cell surface expression of Env and greater gp120 incorporation into nascent virions, a phenotype that we confirmed was not related to altered gp160 processing or Gag-mediated budding, the latter in agreement with another study [49]. Notably, others have also reported mislocalization and increased surface expression of cellular cargos that are trafficked by retromer after Vps26 or Vps35 depletion [18], [25]. Precisely how Env gets relocalized to the plasma membrane in HIV-1 infected cells in the absence of retromer-mediated trafficking is presently unclear since multiple cellular factors may be implicit. However, based on our results we believe that the most likely explanation is that inhibiting retromer-dependent endosome-to-Golgi redirects Env into an alternative trafficking pathway from endosomes towards the plasma membrane, in effect increasing outward Env transport. In support of this we found no evidence of impaired endocytosis of Env in retromer-depleted cells, suggesting that it is altered outward transport, rather than defective internalization that is responsible for increasing Env cell surface expression. The well-established connection between endocytic uptake of proteins and subsequent endosomal sorting leading to plasma membrane recycling makes this scenario likely. Precisely what cellular machinery are involved and how they operate, and whether endosomal recycling compartments that have been recently implicated in Env transport [50] contribute to this warrants future investigation. Interestingly, while our data identifying the retromer complex as new cellular factor interacting with the EnvCT are novel, it is noteworthy that previous proteomics analysis has found Vps35 to be one of many cellular proteins incorporated into viral particles [51]. We have depleted the major isoform Vps26A. Thus it is formally possible that the other isoform Vps26B that localizes to the cytoplasm and plasma membrane, rather than endosomes, may contribute in some way to infectious HIV-1 assembly [52], [53], [54].

Like other viruses, HIV-1 must strike a balance between efficient replication and immune evasion. One way this can be achieved is by controlling exposure of viral proteins to immune surveillance. It is easy to envisage how removing Env (that is not incorporated into virions) from the plasma membrane and recovering it back to the Golgi for subsequent recycling might confer a replicative advantage to the virus. By contrast, it could be argued that limiting Env surface expression and incorporation into virions would be disadvantageous to the virus, not least because we found that virions released from Vps26 depleted cells were more infectious by virtue of increased Env incorporation. Importantly, it is known that increasing surface expression of Env by mutating endocytic motifs within the EnvCT impairs *in vivo* replication of simian viruses in animal models, presumably increasing exposure of Env to antiviral immunity [48]. Since only 8−14 trimeric Env spikes are present on HIV-1 virions and as few as four appear to be needed to mediate attachment and entry of virions to target cells [55], increasing Env cell surface expression and incorporation into virions may not necessarily confer a significant advantage under conditions where infection is already efficient and the virus must strike a balance between robust replication and immune evasion. Given that regulation of Env trafficking to and from the plasma membrane is critical for infectious virus assembly and spread, future studies aimed at identifying the cellular factors controlling Env trafficking and dissecting the molecular details of virus-host interactions is clearly important, not only to better understand how HIV-1 hijacks the host cell machinery, but potentially to inform efforts to manipulate these processes to better expose the virus to antiviral immune responses.

The list of cargo proteins that are trafficked in a retromer-dependent manner is expanding and includes a diverse range of proteins implicated in many cellular processes [19]. We now add the HIV-1 Env glycoprotein to this family of retromer cargos. Importantly this is the first viral structural protein that has been shown to interact with retromer and to be trafficked in a retromer-dependent manner. It seems likely that other viruses may be similarly dependent on retromer for viral protein sorting and replication. While this manuscript was in preparation, Lipovsky et al. identified retromer as a cellular factor implicit in papillomavirus entry [56]. While we found no evidence for retromer playing a role in the initial steps of HIV-1 infection of cells, we provide evidence for retromer during infectious HIV-1 assembly by modulating Env trafficking and virion incorporation, and thus describe the first report of a role for retromer in assembly of a virus. It will clearly be informative to further to investigate the role of other cellular proteins associated with retromer-mediated trafficking, endosomal sorting and recycling in lentiviral replication. In light of the important role that Env plays in lentiviral pathogenesis and anti-viral immunity, illuminating the molecular mechanisms of Env trafficking is timely.

## Materials and Methods

### Cells and viruses

HeLa and HEK293T cells were originally from the ATCC (American Type Culture Collection). HeLa TZM-bl cells were obtained from the Center for AIDS Reagents, National Institutes of Biological Standard and Control, UK (CFAR, NIBSC) and donated by J. Kappes, X. Wu and Tranzyme Inc. Adherent cells were maintained in Dulbecco's Modified Eagle Medium (DMEM) supplemented with streptomycin (100 µg/ml), penicillin (100 U/ml) and 10% fetal calf serum (FCS, Invitrogen). The CD4^+^/CXCR4^+^ T cell line Jurkat CE6.1 and derivative Jurkat line 1G5 (obtained through the AIDS Research and Reference Reagent Program, Division of AIDS, NIAID, NIH: from Dr. Estuardo Aguilar-Cordova and Dr. John Belmont) were maintained in RPMI 1640 supplemented with streptomycin (100 µg/ml), penicillin (100 U/ml) and 10% fetal calf serum (Invitrogen). The HIV-1 clone pNL4.3 was produced by Dr Malcolm Martin and obtained from the NIH AIDS Reagent and Reference Program (NIAID, NIH, USA). The CTdel-144 mutant (referred to herein a Δ144) was a kind gift from Dr Eric Freed (National Cancer Institute, Frederick, USA) [36], [57]. Stocks of infectious virus were made by transfecting 293T cells using Fugene 6 (Promega) and infectious viral titer measured on HeLa TZM-bl reporter cells using the Bright-Glo Luciferase assay kit (Promega).

### Plasmids and transfections

The CMS28 retroviral expression vector, a derivative of MIGR1, was used in which the BglII/XhoI/HpaI/EcoI polylinker had been replaced with EcoRI/NotI/Xhol (a gift from M.Malim King's College London). The CD8-CIMPR fusion construct generated by M. Seaman [18] was subcloned into CMS28 and was a gift from S. Neil (King's College London). The CD8-gp41 fusion constructs containing the ecto and transmembrane domains of CD8 fused to the cytoplasmic tail of HIV-1 gp41 were generated as follows. CD8 DNA was PCR amplified from the CD8-CIMPR using forward (5′-ATAGAATTCATGGCCTTACCAGTGA-3′) and reverse (5′-ATAGCGGCCGCGGTGATAACCAGT-3′) primers containing an EcoRI and NotI restriction site respectively. CD8 PCR products were ligated into CMS28 to generate CMS28-CD8. The full-length gp41 cytoplasmic tail was PCR amplified from the HIV-1 molecular clone pNL4.3 using a forward primer (5′-AAGCGGCCGCAAATAGAGTTAGGCAG-3′) containing a NotI restriction site and a reverse primer (5′-ATCTCGAGTTATAGCAAAATCCTTTCCAA-3′) containing a XhoI restriction site. The gp41 forward primer was designed such that all six amino acids upstream of the YSPL endocytosis motif in the gp41 cytoplasmic tail were included in addition to an alanine triplet encoded by the primer, maintaining this YSPL motif within the required distance from the plasma membrane for endocytosis [58]. For PCR amplification of the truncated gp41 cytoplasmic tails the reverse primers (5′-ATCTCGAGTTATAGTTCCTGACTCCAATACTG-3′ or 5′-ATCTCGAGTTAAAGTGCTAAGGATCCGTTCA-3′) containing a XhoI site and 3′ stop codon were used, terminating immediately after L805 and L753 in NL4.3 respectively (corresponding to L807 and L755 in HxB2). HIV-1 gp41 PCR products were ligated into CMS28-CD8 to generate 3 constructs: CD8-gp41CT, CD8-gp41L805* and CD8-gp41L753*. To generate ΔIS1 and ΔIS2, site-directed mutagenesis was used to delete residues V747-S760 (inclusive) or L761-L783 (inclusive) from the CD8-gp41CT plasmid.

All constructs were verified by sequencing and stable expression in HeLa cells was achieved using puromycin selection and confirmed by western blotting and flow cytometry.

### RNAi knockdown

Adherent cells were seeded at a density of 3×10^5^ per well and transfected with Dharmacon Smartpool targeting human Vps26A, Vps35 or a non-targeting control siRNA using Oligofectamine based on the method of Seaman [18]. Unless otherwise stated, 200 nM of siRNA was used. Twenty-four hours later the cells were reseeded and the following day an identical second transfection was performed. Forty-eight hours later the cells were harvested and Vps26 and Vps35 knockdown were determined by western blotting. HeLa TZM-bl cells were infected with pNL4.3 or pNL4.3-Δ144 virus at an MOI of 0.1 immediately after the second knockdown. Excess virus was removed by washing and cells incubated at 37°C for 48 h prior to analysis. RNAi knockdown of Vps26 in T cells was performed by generating 4 individual oligonucleotide hairpins based on the Dharmacon Smartpool target sequences. shRNA hairpins were cloned individually into the HIV-1 vector pCSRQ, a modified version of pSIREN RetroQ [59] (a gift from G. Towers, UCL) and co-transfected into 293T cells with the VSV-G envelope plasmid. Virions were used to infect T cells and stable cell lines selected with puromycin.

### Virus release assay

siRNA transfected HeLa cells or shRNA expressing Jurkat T cells were infected with HIV-1 and incubated for 48 h. Virus was harvested from supernatants by filtration and equal volumes were titrated on Jurkat 1G5 cells and infectivity measured by luciferase assay (Bright-Glo Promega). HIV-1 Gag p24 ELISA was performed as described [60]. For biochemical analysis by western blotting, virus-containing supernatants were ultracentrifuged through a 25% sucrose cushion and pelleted virus was resuspended in PBS and stored at -80°C. Virus was lysed in SDS-PAGE loading buffer and analyzed by SDS-PAGE and western blotting. Cells were pelleted, washed in cold PBS and lysed in RIPA buffer on ice for 10 min. Soluble proteins were collected following centrifugation at 15 000×g for 10 min at 4°C and the protein concentration was determined using a BCA Protein Assay Reagent Kit (Pierce, USA).

### Western blotting

Twenty µg of cell lysate and an equal volume of purified virus were separated by SDS-PAGE and analyzed by western blotting using the following primary antibodies: rabbit antisera raised against HIV-1 Gag (donated by Dr G. Reid and obtained from the CFAR); rabbit antisera raised against gp120 (donated by Dr S. Ranjbar and obtained from the CFAR); human anti HIV-1 gp41 Mab 246-D (donated by Dr S. Zollar-Pazner and Dr M. Gorny, obtained from the CFAR); rabbit anti-Vps26 (Abcam); rabbit anti-Vps35 (Abcam); rabbit anti-actin (Sigma); mouse anti-tubulin (DM1A, Sigma); anti-CD8 (sc-7188, Santa Cruz). Primary antibodies were detected with goat anti-rabbit or anti-mouse HRP (DAKO) and visualized by ECL (GE Healthcare). To arrest protein synthesis cells were washed and incubated in media containing 100 µg/ml cycloheximide or carrier control for up to 6 h. Cells were detached with EDTA, lysed in RIPA buffer and analyzed by SDS-PAGE and Western blotting.

### Flow cytometry

Cells were washed in cold FACS wash buffer (FWB: PBS with 1% FCS and 0.01% sodium azide) and incubated on ice for 1 h with 20 µg/ml of the anti-gp120 mAb 2G12 (Polymun) to detect cell surface expressed HIV-1 Env or a mAb specific for CD8 (UCHT-4, Abcam). Cells were subsequently washed in cold FWB, fixed in 4% formaldehyde and incubated with anti-human or anti-mouse IgG-phycoerythrin for 30 min. Acquisition and analysis was performed using a Becton Dickinson FACS Calibur.

### Immunofluorescence microscopy

Adherent cells were detached with 2 mM EDTA in PBS and reseeded onto glass coverslips 24 h prior to staining. Cells were fixed in 4% formaldehyde in PBS-1% BSA and permeabilized either in 0.1% Triton X-100/5% FCS for 20 min at RT or cold 100% methanol for 5 minutes. Intracellular staining used the following primary antibodies: rabbit anti-Vps26 serum (Abcam), mouse anti-EEA1 mAb (clone 14, BD Biosciences), mouse anti-Lamp1 ascites (H4A3, Developmental Studies Hydridoma Bank, University of Iowa), mouse anti-cation independent mannose 6 phosphate receptor mAb (2G11, Abcam), mouse anti-CD8 mAb (UCHT-4, Abcam) and mouse anti-giantin mAb (9B6, Abcam). HIV-1 Gag was detected with rabbit antisera against Gag p17 and p24 (donated by G. Reid and obtained from the CFAR, NIBSC) and HIV-1 Env with the human mab 2G12 (Polymun). Primary antibodies were detected with either FITC, TRITC, Cy5 (Jackson Immunoresearch) or Alexa-conjugated (Invitrogen) anti-mouse, anti-human or anti-rabbit secondary antibodies that were tested for an absence of inter-species reactivity. Anti-mouse isotype-specific secondary antibodies were also used that were tested for an absence of inter-isotype reactivity (Invitrogen). Coverslips were mounted with ProLong antifade mounting solution containing DAPI (Invitrogen) and cells were imaged through a 63×1.4 NA oil immersion lens with an inverted Olympus IX71 microscope (DeltaVision ELITE Image Restoration Microscope, Applied Precision) and a CoolSNAP HQ2 camera. Images were acquired as serial 0.2 µm sections through the entire volume of the cell and deconvolved with softWoRx 5.0. Processing was performed using Huygens Professional version 4.0 and Adobe Photoshop C3.

### Colocalization analysis

Quantification of colocalization was performed using the DeltaVision softWoRx image acquisition and analysis software. Z stacks of 0.2 µm sections were acquired for each fluorescence channel through the entire volume of the cell and images deconvolved using softWoRx. To quantify the degree of colocalization, the Pearson correlation coefficient (R) values were calculated using the softWoRx colocalization module. To do this, a colocalized image is generated using the two selected channels. Scatter plots are plotted showing the pixel-by-pixel intensity of the two fluorescent channels and the R value is calculated by dividing the covariances of each channel by their standard deviations. Colocalization analysis was performed on a single *xy* slice through the middle of the cell (determined by DAPI staining of the nucleus). At least twenty cells selected at random from a total of 3 independent experiments were analysed. R values reported are the mean and SEM.

### Antibody uptake and trafficking assay

For immunofluorescence staining of CD8-fusion proteins endocytosed from the plasma membrane an antibody-feeding method was used. To label plasma membrane Env or CD8 cells were cooled on ice, washed in ice cold DMEM and incubated with 1 µg/ml anti-CD8 mAb for 30 minutes on ice. Excess antibody was removed by washing with ice cold DMEM and the cells were incubated at 37°C for up to 120 minutes to allow antibody uptake. Cells were then fixed, permeabilized and stained with primary antibodies for cellular markers, followed by secondary antibodies as described above.

### Native co-immunoprecipitations

Native coimmunoprecipitations of CD8-CIMPR and CD8-gp41CT proteins was performed essentially as described previously [25]. The method was adapted to use 9×10^6^ untransfected HeLa cells and HeLa cells expressing CD8-gp41 proteins and 4.5×10^6^ cells expressing the CI-MPR. Lysates were precleared by addition of 1.5 mg Protein G Dynabeads (Life Technologies) for 60 min at 4°C. Lysates were then incubated with 1.5 mg Protein G Dynabeads precoupled with 5 µg mouse anti-CD8 (clone UCHT4) for 90 min at 4°C. Samples were washed 3 times with lysis buffer [25], eluted in SDS sample buffer and analyzed by SDS-PAGE and western blotting using rabbit anti-Vps35, rabbit anti-Vps26 and rabbit anti-CD8 (Santa Cruz, sc-7188).

### Protein expression and purification

GST and GST-CIMPR-CT were a gift from M. Tabuchi and F. Kishsi(Kagawa University, Japan) [26]. To construct the GST-gp41CT, restriction enzyme sites in GST-CIMPR-CT were replaced using site-directed mutagenesis, the CIMPR-CT was removed and the gp41CT inserted and verified by sequencing. GST-fusion proteins were expressed in Rosetta-gami 2(DE3)pLysS cells (Novagen) and purified as previously described [26] with modifications. Briefly, GST and GST-CIMPR-CT were induced with IPTG according to the method of Tabuchi *et al*., [26] and GST-gp41-CT protein according to Wyss *et al*., [12]. Following induction, cells were pelleted, subjected to five freeze/thaw cycles with liquid nitrogen, resuspended in buffer A (0.5% Triton X-100 in PBS containing 1 mM phenylmethylsulfonyl fluoride (PMSF) and 1 mg/mL lysozyme), and disrupted by sonication. Lysates were cleared by centrifugation and GST-tagged proteins were purified using glutathione-Sepharose 4B beads (GE Healthcare) overnight at 4°C with rotation. Beads were then washed and proteins eluted with buffer G (20 mM reduced glutathione, 0.1% v/v Triton X-100, 50 mM Tris-HCl, pH 9.0). GST-gp41-CT protein was subjected to an additional concentration and purification step using 50% v/v of saturated (NH4)_2_SO_4_. All purified GST and GST-fusion proteins were dialysed overnight in PBS and protein concentrations determined using a BCA protein assay kit (Pierce).

FLAG-tagged retromer complex (a gift from M. Tabuchi and F. Kishsi (Kagawa University, Japan) was expressed and purified as described, with minor modifications [26]. Briefly, Rosetta-gami 2(DE3)pLysS containing the pET23d-3 × FLAG-Retromer construct were induced with IPTG [26] and cell pellets resuspended in buffer B (50 mM NaH2PO4, 300 mM NaCl, 1 mM imidazole, pH 8.0, containing complete EDTA-free protease inhibitor cocktail [Roche Molecular Biochemicals], 1 mM PMSF and 1 mg/mL lysozyme). Cells were freeze-thawed using liquid nitrogen, sonicated, and lysates were cleared by centrifugation before being applied to a Ni-NTA agarose column (Qiagen). Columns were then washed (50 mM NaH_2_PO_4_, 300 mM NaCl, 5 mM imidazole, pH 8.0) and proteins eluted (50 mM NaH_2_PO_4_, 300 mM NaCl, 250 mM imidazole, pH 8.0). Eluted 3 × FLAG-Retromer complex was dialysed overnight in PBS and protein concentration determined using a BCA protein assay kit (Pierce).

### GST pull-down assay

GST pulldowns were performed as described [26] except that proteins were eluted at 4°C with rotation in modified buffer G (20 mM glutathione, 0.1% v/v Triton X-100, 50 mM Tris-HCl, pH 9.0). Samples were analyzed by SDS-PAGE and western blotting using anti-Vps35 and anti-Vps26 as described above.

### Mass spectrometry

Immunoprecipitated samples were separated by SDS-PAGE and gel bands corresponding to the known size of retromer components were excised, digested with trypsin and peptides analyzed mass spectrometry using an Orbitrap XL (Thermo Electron, Bremen, Germany) and identified using Mascot v2.4 (Matrix Science) and SwissProt databases.

### Statistical analysis

Statistical significance was calculated either using the parametric Anova test for multiple comparisons with Bonferroni correction or parametric student's *t* test. Significance was assumed when *p*<0.05.

## Supporting Information

Figure S1 **Effect of Vps26 knockdown on steady-state CIMPR localization.** HeLa TZM-bl cells were left untreated or transfected with siRNA targeting Vps26, fixed, permeabilized and stained for endogenous CIMPR (green), early endosomal marker EEA1 (red) or Vps26 (red). Panels are single *xy* slices and are representative examples from three independent experiments. Scale bar is 20 microns.
(TIF)

Figure S2 **A) Vps26 knockdown does not inhibit initial HIV-1 infection.** HIV-1 infected HeLa TZM-bl cells from Figure 1 were assayed for expression of the HIV-1 Tat driven luciferase reporter gene 24 h after infection. Infectivity is expressed as relative light units (RLU). Error bars show the SD from a representative experiment. **B) HIV-1 infection alone does not alter Vps26 and Vps35 expression.** HeLa TZM-bl cells were either left untreated or infected with HIV-1 WT NL4.3 or NL4.3Δ144. Cell lysates were subjected to SDS-PAGE and western blotting for Vps26, Vps35 and tubulin. One representative of two independent experiments is shown. **C) Infection with VSV-G pseudotyped HIV-1 does not alter Vps26 and Vps35 expression.** HeLa TZM-bl cells were either left untreated or infected with VSV-G pseudotyped HIV-1 WT NL4.3. Cell lysates were subjected to SDS-PAGE and western blotting for Vps26, Vps35 and tubulin. One representative of two independent experiments is shown.
(TIF)

Figure S3 **Colocalization of Env with EEA1 and Lamp1 following Vps26 depletion.** HeLa TZM-bl cells were infected with HIV-1, fixed, permeabilized and stained for HIV-1 Env (green) and the early endosome marker EEA1 or late endosome/lysosome marker Lamp1 (red). Panels are single *xy* slices and are representative examples from three independent experiments. Scale bar is 20 microns. The amount of immunoreactive Env colocalizing with EEA1 (R value for UT  = 0.20+/−0.05; Vps26 KD  = 0.21+/−0.03) and Lamp1 (R value for UT  = 0.22+/−0.02; Vps26 KD  = 0.27+/−0.04) was calculated from at least 20 cells.
(TIF)

Figure S4 **Immunofluorescence localization of HIV-1 Gag in control or Vps26 siRNA treated cells.** HeLa TZM-bl cells were treated with siRNA against Vps26, infected with HIV-1, fixed, permeabilized and stained for HIV-1 Gag (red) and the late endosome/lysosome marker Lamp1 or retromer component Vps26 (green). Panels are single *xy* slices and are representative examples from three independent experiments. The amount of immunoreactive Gag colocalized with Lamp1 (R value for UT  = 0.06+/−0.01; Vps26 KD  = 0.1+/−0.02) and Vps26 (R value for UT  = 0.1+/−0.02; Vps26 KD  = 0.04+/−0.01) was calculated from at least 20 cells.
(TIF)

Figure S5 **CD8-gp41 cytoplasmic tail constructs.** **A)** The CD8 ecto and transmembrane domains were ligated to the gp41 cytoplasmic tail of Env, separated by an AAA linker. CD8-gp41CT contains the entire gp41 cytoplasmic tail of HIV-1 strain NL4.3 including the native stop codon after the C terminal leucine. CD8-gp41-L805* and CD8-gp41-L753* terminate where indicated. Constructs were cloned into CMS28 and transfected to generate stably-expressing HeLa cells. **B)** Western blot showing expression of appropriately sized CD8 fusion constructs. Cell lysates were separated by SDS-PAGE and western blotting was performed with an anti-CD8 antibody. Tubulin serves as a loading control.
(TIF)

Figure S6 **HeLa cells not expressing CD8-fusion proteins do not internalize anti-CD8 by non-specific fluid phase uptake.** Untransfected HeLa cells were used for antibody-feeding assays using anit-CD8 as described in Figure 4 and 5.
(TIF)

Figure S7 **Mass spectrometry (MS) identification of Vacuolar protein sorting-associated protein 26A (VPS 26A) from CD8-gp41CT.** **A)** Peptide EITGIGPSTTTETETIAK (amino acid position 215 to 232) of Vps26A was identified by MS and further confirmed by fragmentation via MS/MS. **B)** Sixteen of the 18 amino acids were identified after MS/MS fragmentation of the peptide (a minimum of 8 consecutive amino acids in a peptide are sufficient for protein identification).
(TIF)
