# Supplementary figures and images for: Retromer Regulates HIV-1 Envelope Glycoprotein Trafficking and Incorporation into Virions
Source: PLoS Pathog. 2014 Nov 13;10(11):e1004518. doi: 10.1371/journal.ppat.1004518 (PMC4231165; doi:10.1371/journal.ppat.1004518)

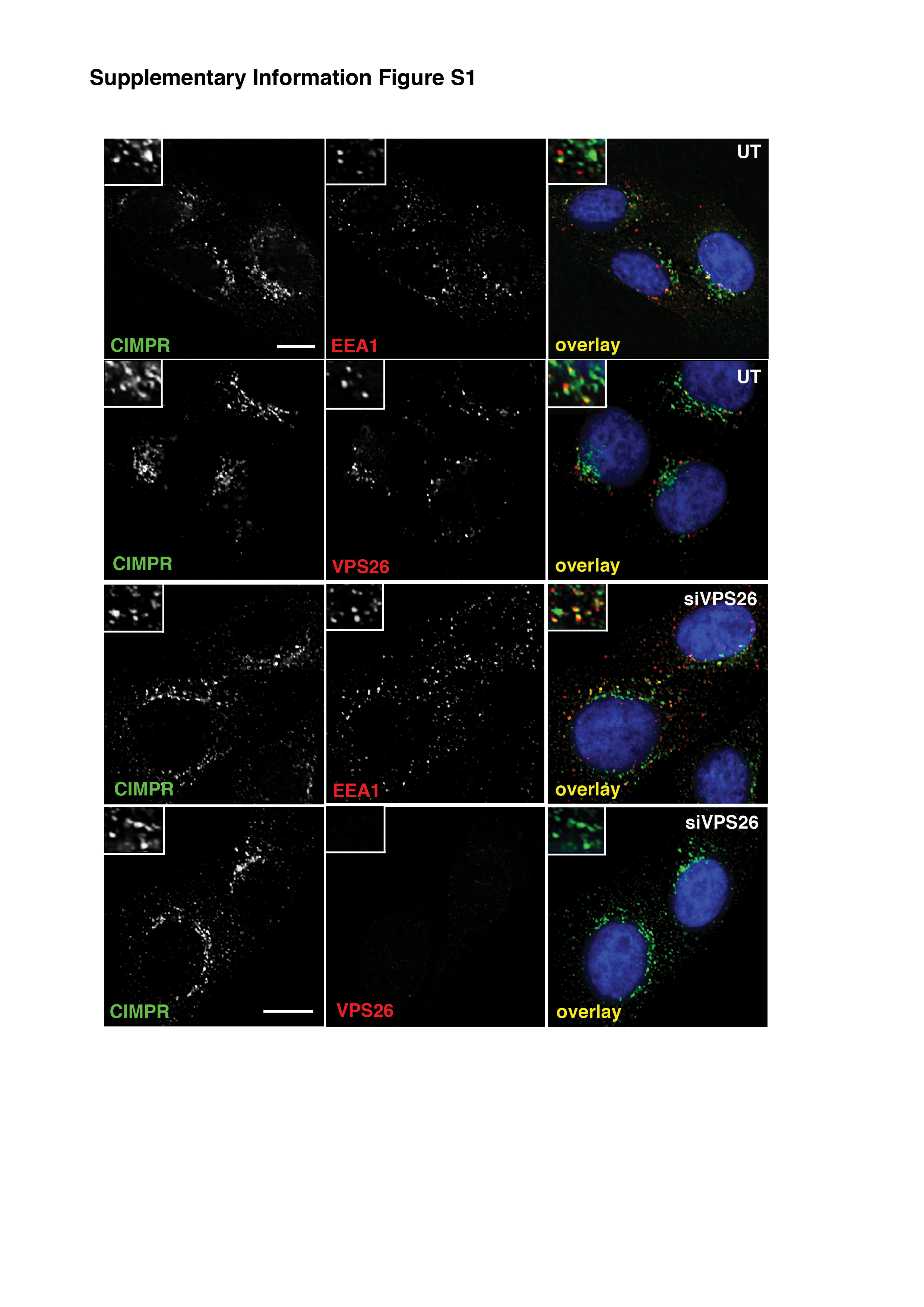

Supplement: Figure S1 — Effect of Vps26 knockdown on steady-state CIMPR localization. HeLa TZM-bl cells were left untreated or transfected with siRNA targeting Vps26, fixed, permeabilized and stained for endogenous CIMPR (green), early endosomal marker EEA1 (red) or Vps26 (red). Panels are single xy slices and are representative examples from three independent experiments. Scale bar is 20 microns. (TIF) [file ppat.1004518.s001.tif]

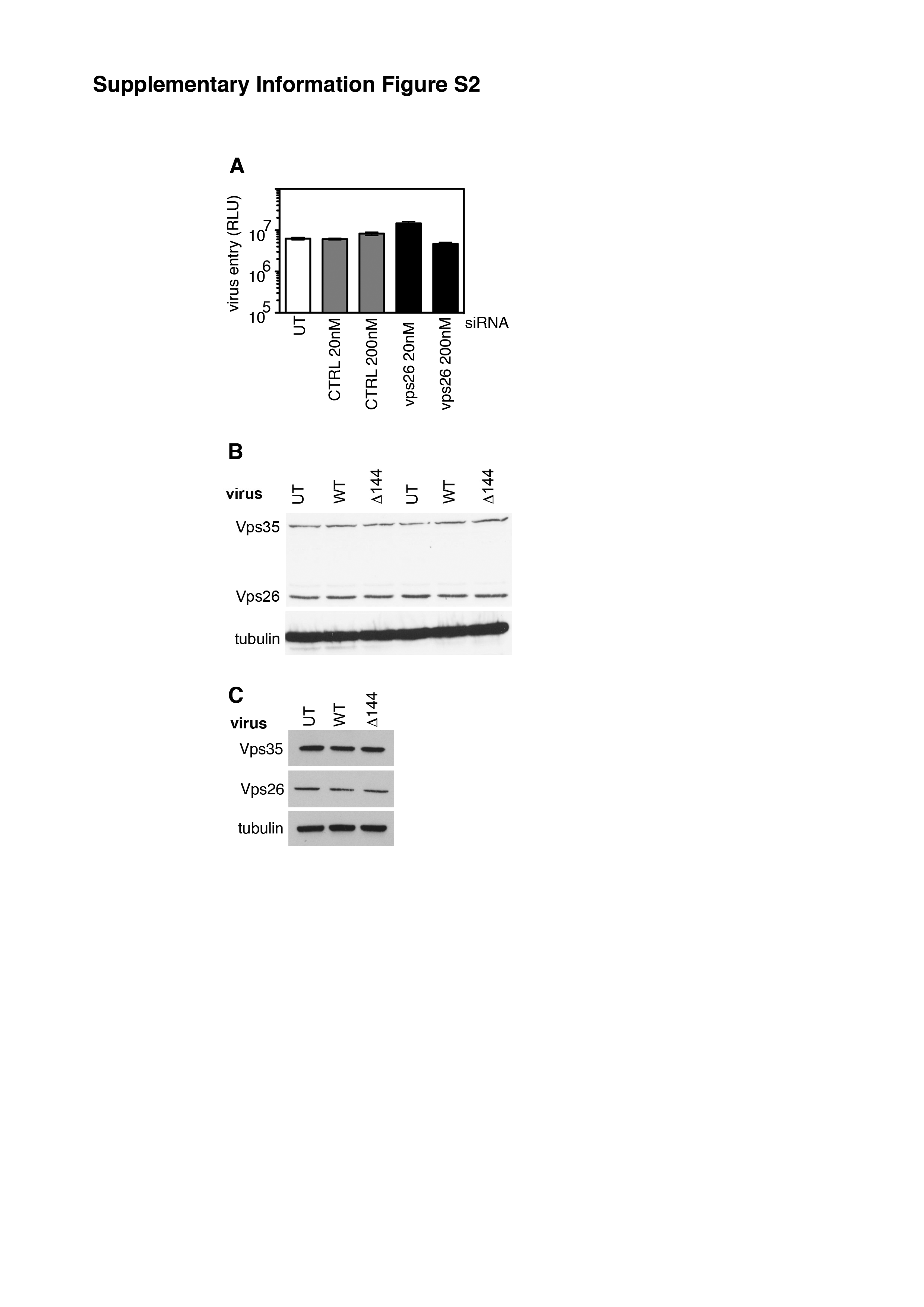

Supplement: Figure S2 — A) Vps26 knockdown does not inhibit initial HIV-1 infection. HIV-1 infected HeLa TZM-bl cells from Figure 1 were assayed for expression of the HIV-1 Tat driven luciferase reporter gene 24 h after infection. Infectivity is expressed as relative light units (RLU). Error bars show the SD from a representative experiment. B) HIV-1 infection alone does not alter Vps26 and Vps35 expression. HeLa TZM-bl cells were either left untreated or infected with HIV-1 WT NL4.3 or NL4.3Δ144. Cell lysates were subjected to SDS-PAGE and western blotting for Vps26, Vps35 and tubulin. One representative of two independent experiments is shown. C) Infection with VSV-G pseudotyped HIV-1 does not alter Vps26 and Vps35 expression. HeLa TZM-bl cells were either left untreated or infected with VSV-G pseudotyped HIV-1 WT NL4.3. Cell lysates were subjected to SDS-PAGE and western blotting for Vps26, Vps35 and tubulin. One representative of two independent experiments is shown. (TIF) [file ppat.1004518.s002.tif]

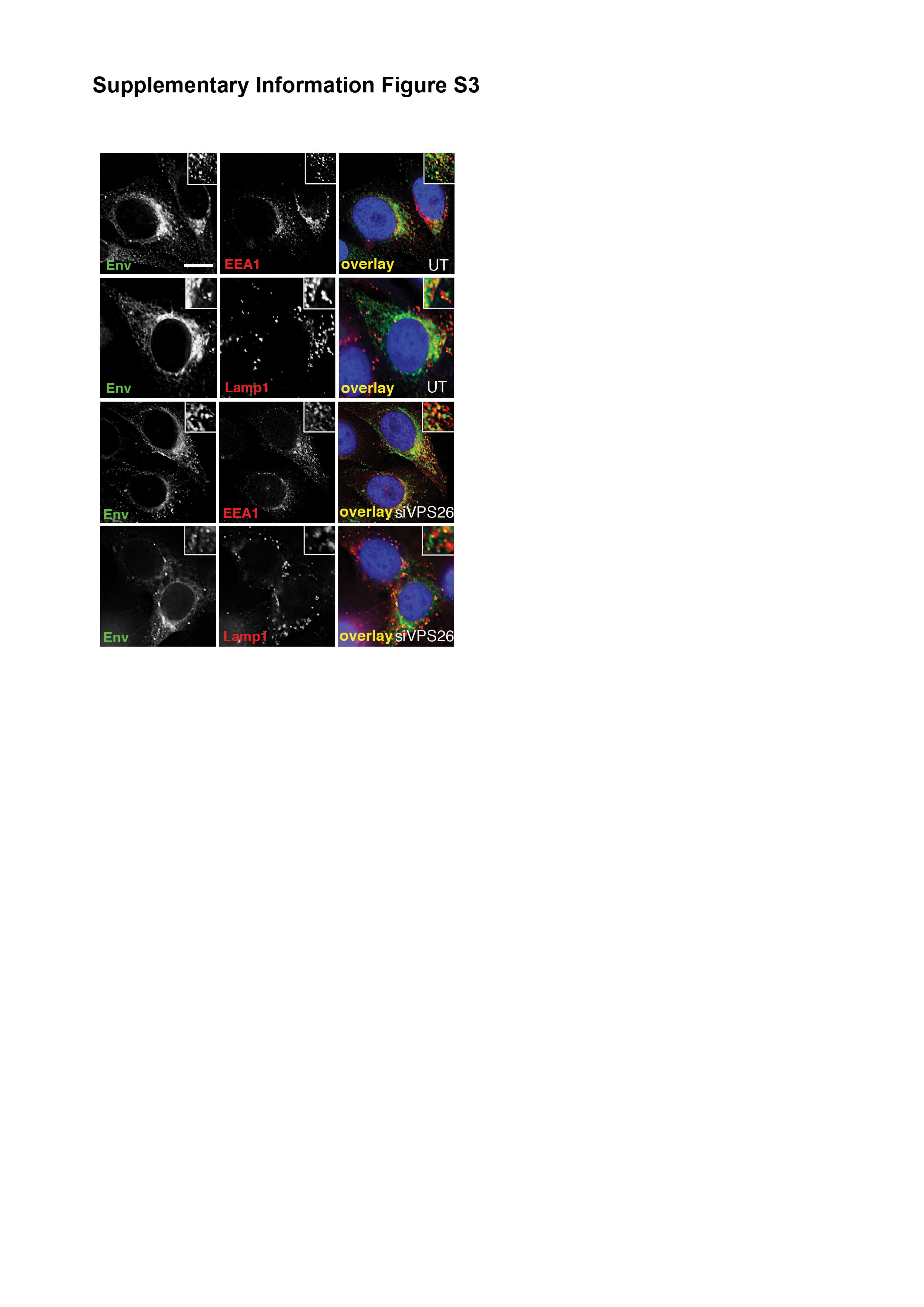

Supplement: Figure S3 — Colocalization of Env with EEA1 and Lamp1 following Vps26 depletion. HeLa TZM-bl cells were infected with HIV-1, fixed, permeabilized and stained for HIV-1 Env (green) and the early endosome marker EEA1 or late endosome/lysosome marker Lamp1 (red). Panels are single xy slices and are representative examples from three independent experiments. Scale bar is 20 microns. The amount of immunoreactive Env colocalizing with EEA1 (R value for UT = 0.20+/−0.05; Vps26 KD = 0.21+/−0.03) and Lamp1 (R value for UT = 0.22+/−0.02; Vps26 KD = 0.27+/−0.04) was calculated from at least 20 cells. (TIF) [file ppat.1004518.s003.tif]

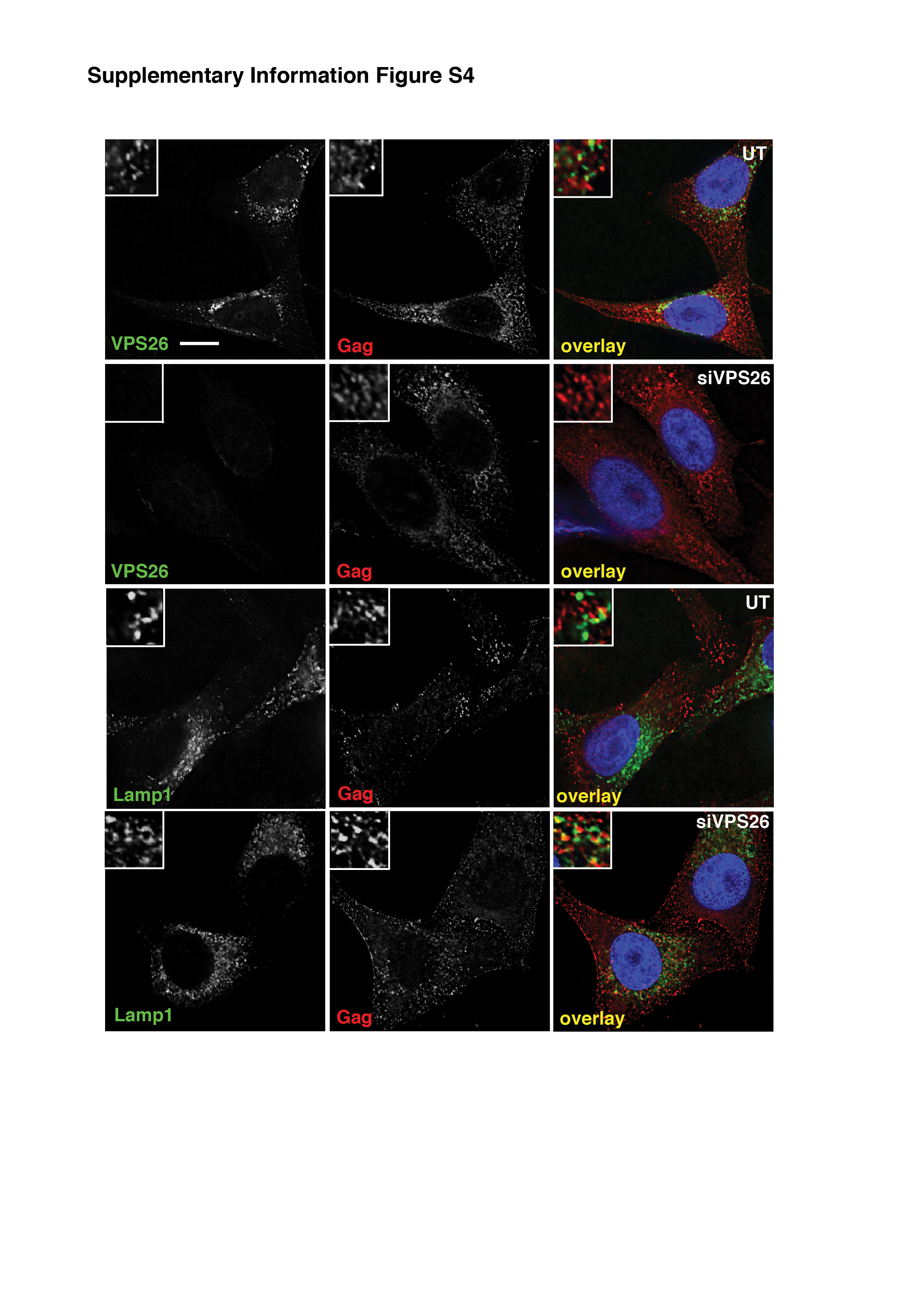

Supplement: Figure S4 — Immunofluorescence localization of HIV-1 Gag in control or Vps26 siRNA treated cells. HeLa TZM-bl cells were treated with siRNA against Vps26, infected with HIV-1, fixed, permeabilized and stained for HIV-1 Gag (red) and the late endosome/lysosome marker Lamp1 or retromer component Vps26 (green). Panels are single xy slices and are representative examples from three independent experiments. The amount of immunoreactive Gag colocalized with Lamp1 (R value for UT = 0.06+/−0.01; Vps26 KD = 0.1+/−0.02) and Vps26 (R value for UT = 0.1+/−0.02; Vps26 KD = 0.04+/−0.01) was calculated from at least 20 cells. (TIF) [file ppat.1004518.s004.tif]

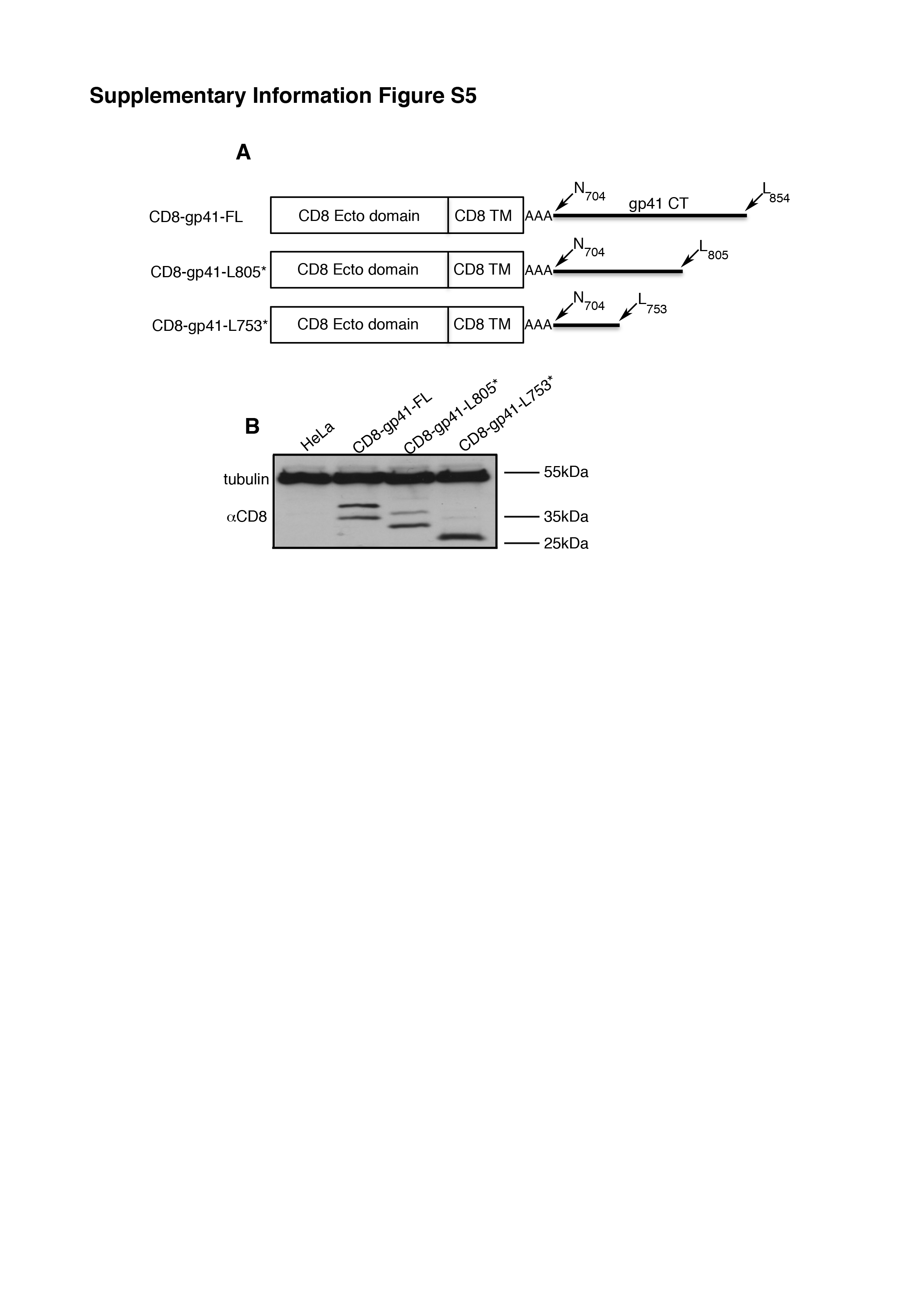

Supplement: Figure S5 — CD8-gp41 cytoplasmic tail constructs. A) The CD8 ecto and transmembrane domains were ligated to the gp41 cytoplasmic tail of Env, separated by an AAA linker. CD8-gp41CT contains the entire gp41 cytoplasmic tail of HIV-1 strain NL4.3 including the native stop codon after the C terminal leucine. CD8-gp41-L805* and CD8-gp41-L753* terminate where indicated. Constructs were cloned into CMS28 and transfected to generate stably-expressing HeLa cells. B) Western blot showing expression of appropriately sized CD8 fusion constructs. Cell lysates were separated by SDS-PAGE and western blotting was performed with an anti-CD8 antibody. Tubulin serves as a loading control. (TIF) [file ppat.1004518.s005.tif]

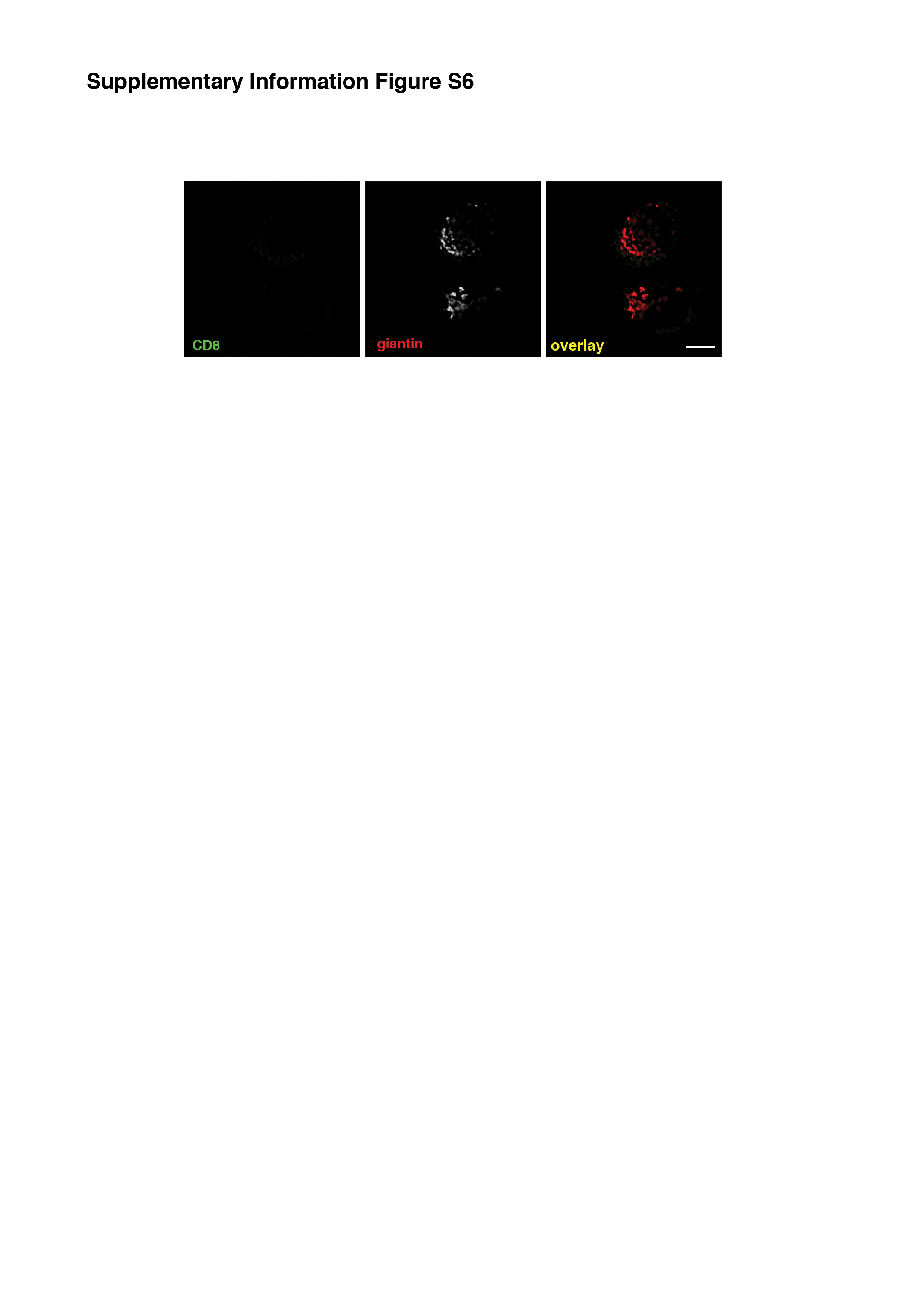

Supplement: Figure S6 — HeLa cells not expressing CD8-fusion proteins do not internalize anti-CD8 by non-specific fluid phase uptake. Untransfected HeLa cells were used for antibody-feeding assays using anit-CD8 as described in Figure 4 and 5. (TIF) [file ppat.1004518.s006.tif]

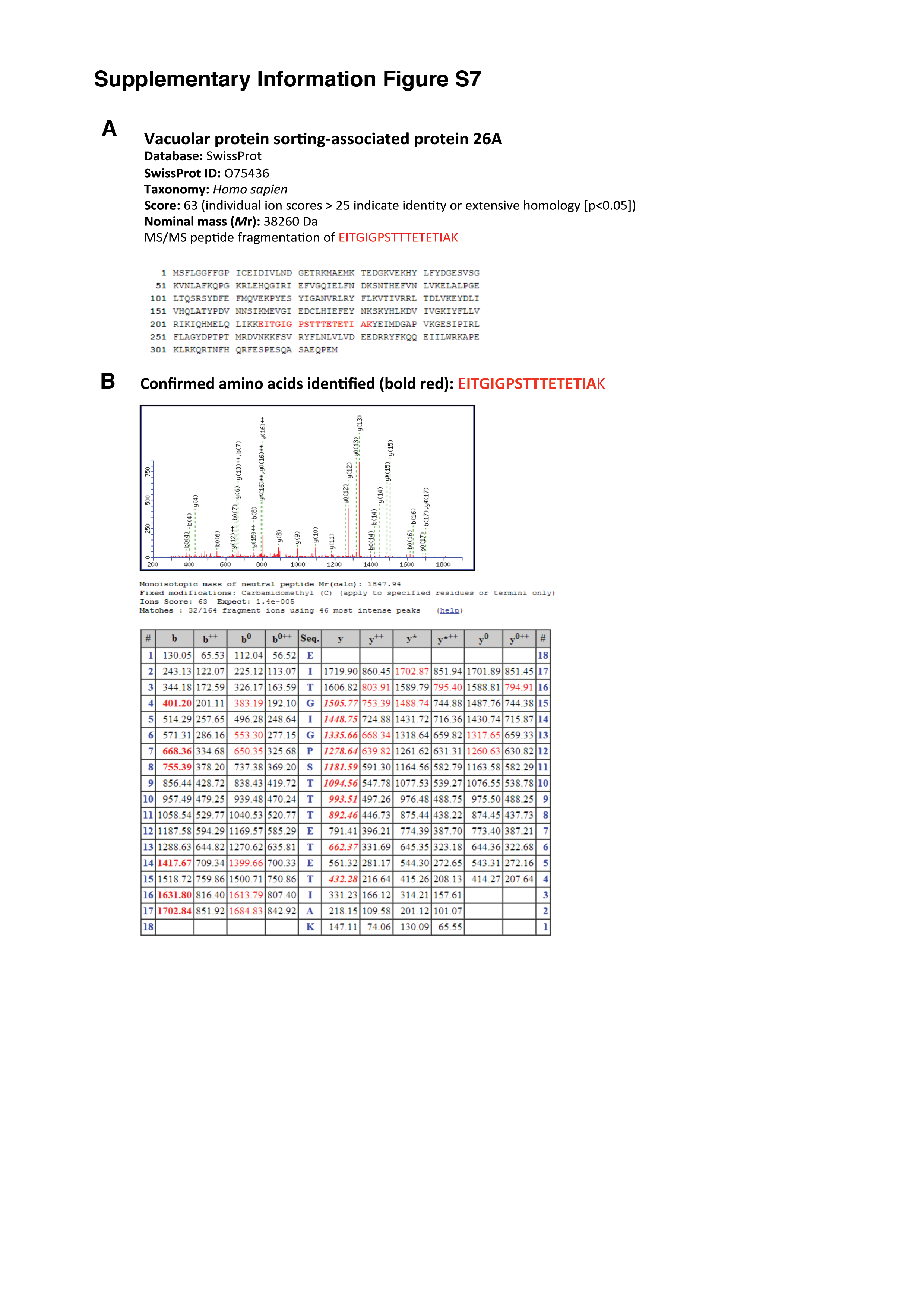

Supplement: Figure S7 — Mass spectrometry (MS) identification of Vacuolar protein sorting-associated protein 26A (VPS 26A) from CD8-gp41CT. A) Peptide EITGIGPSTTTETETIAK (amino acid position 215 to 232) of Vps26A was identified by MS and further confirmed by fragmentation via MS/MS. B) Sixteen of the 18 amino acids were identified after MS/MS fragmentation of the peptide (a minimum of 8 consecutive amino acids in a peptide are sufficient for protein identification). (TIF) [file ppat.1004518.s007.tif]
